# A membrane protein of the rice pathogen *Burkholderia glumae* required for oxalic acid secretion and quorum sensing

**DOI:** 10.1111/mpp.13376

**Published:** 2023-07-10

**Authors:** Asif Iqbal, George Nwokocha, Vijay Tiwari, Inderjit K. Barphagha, Anne Grove, Jong Hyun Ham, William T. Doerrler

**Affiliations:** ^1^ Department of Biological Sciences Louisiana State University Baton Rouge Louisiana USA; ^2^ Department of Plant Pathology and Crop Physiology Louisiana State University Agricultural Center Baton Rouge Louisiana USA

**Keywords:** bacterial panicle blight, oxalic acid, pH homeostasis, quorum sensing

## Abstract

Bacterial panicle blight is caused by *Burkholderia glumae* and results in damage to rice crops worldwide. Virulence of *B. glumae* requires quorum sensing (QS)‐dependent synthesis and export of toxoflavin, responsible for much of the damage to rice. The DedA family is a conserved membrane protein family found in all bacterial species. *B. glumae* possesses a member of the DedA family, named DbcA, which we previously showed is required for toxoflavin secretion and virulence in a rice model of infection. *B. glumae* secretes oxalic acid as a “common good” in a QS‐dependent manner to combat toxic alkalinization of the growth medium during the stationary phase. Here, we show that *B. glumae* Δ*dbcA* fails to secrete oxalic acid, leading to alkaline toxicity and sensitivity to divalent cations, suggesting a role for DbcA in oxalic acid secretion. *B. glumae* Δ*dbcA* accumulated less acyl‐homoserine lactone (AHL) QS signalling molecules as the bacteria entered the stationary phase, probably due to nonenzymatic inactivation of AHL at alkaline pH. Transcription of toxoflavin and oxalic acid operons was down‐regulated in Δ*dbcA*. Alteration of the proton motive force with sodium bicarbonate also reduced oxalic acid secretion and expression of QS‐dependent genes. Overall, the data show that DbcA is required for oxalic acid secretion in a proton motive force‐dependent manner, which is critical for QS of *B. glumae*. Moreover, this study supports the idea that sodium bicarbonate may serve as a chemical for treatment of bacterial panicle blight.

## Graphical abstract

The quorum sensing‐regulated synthesis of oxalic acid by ObcAB is needed to prevent alkaline degradation of C8‐HSL quorum sensing signalling molecules and avoid alkaline toxicity. The DedA family protein DbcA is required for oxalic acid secretion.

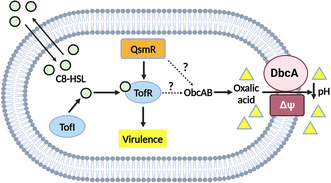

## 1 INTRODUCTION

*Burkholderia glumae* is the major cause of bacterial panicle blight (BPB) disease in rice. *B. glumae* is a seedborne pathogen that causes BPB in rice (Ham et al., 2011). *B. glumae* can endophytically grow inside the rice seed, migrate to upper tissues and leaves during plant growth, and infect the rice panicle during the booting stage (Naughton et al., 2016; Ortega & Rojas, 2021). Hot and humid conditions are favourable for the disease (Shew et al., 2019). Therefore, global warming may increase the occurrence of the disease across the world. BPB can reduce rice production by 75% in severely infected fields (Ham et al., 2011). Furthermore, global rice production needs to increase by 26% by 2035 to meet the rice demand for Earth's growing population (White et al., 2020). While important research has been conducted to understand the virulence of *B. glumae* (Chen et al., 2012; Kim et al., 2004; Lelis et al., 2019), no chemical treatment has been adopted to control BPB in rice. Some rice varieties have shown reduced sensitivity to BPB; however, no rice variety has shown complete resistance to BPB (Ham et al., 2011). Therefore, there is an urgent need to find a solution to control BPB in rice to protect global rice production.

The occurrence of BPB due to *B. glumae* infection is a multifactorial process that includes several virulence factors, including toxoflavin (Ham et al., 2011). Toxoflavin, a 7‐azapteridine antibiotic, is the major virulence factor of *B. glumae* and mainly responsible for the symptoms of BPB in the rice plant. Toxoflavin acts as an electron carrier between oxygen and NADH and is able to produce hydrogen peroxide, increasing the levels of reactive oxygen species, leading to toxicity to the plant (Latuasan & Berends, 1961; Park et al., 2019). *B. glumae* synthesizes and transports toxoflavin by activating the transcription of the *tox* operons in a process controlled by quorum sensing (QS) (Chen et al., 2012; Kim et al., 2004; Suzuki et al., 2004).

QS is a cell‐to‐cell communication system enabling bacteria to control gene expression and behaviours in a cell density‐dependent manner (Abisado et al., 2018; Papenfort & Bassler, 2016; Rattray et al., 2022). *B. glumae* regulates its virulence factors in a QS‐dependent manner mediated by TofI and TofR (homologues of *Vibrio fischeri* LuxI and LuxR, respectively) (Chen et al., 2012; Kim et al., 2004). TofI produces two types of QS signalling molecules, *N*‐hexanoyl homoserine lactone (C6‐HSL) and *N*‐octanoyl homoserine lactone (C8‐HSL) (Kim et al., 2004). C8‐HSL specifically binds to the receptor TofR to regulate QS‐dependent gene expression, while the function of C6‐HSL is unknown (Chen et al., 2012; Kim et al., 2004). The TofR–C8‐HSL complex activates the expression of the ToxJ regulatory protein, which in turn activates the expression of ToxR, a LysR‐type transcriptional regulator. ToxR binds to the promoters of the *toxABCDE* and *toxFGHI* operons and activates the transcription of toxoflavin biosynthesis and transporter genes. The TofR–C8‐HSL complex also regulates protease activity, flagellum biogenesis, and flagellar motility in *B. glumae* (Ham et al., 2011). Interference with QS is a promising approach to treat or prevent plant diseases caused by bacteria (Helman & Chernin, 2015).

Oxalic acid is a well‐known metabolite produced by bacteria, fungi, plants, and animals (Nakata, 2011). The functional role of oxalic acid is species‐specific. In bacteria and fungi, oxalic acid plays several important roles, contributing to metal tolerance, nutrient acquisition, and virulence (Gadd, 1999; Hamel et al., 1999; Munir et al., 2001). Production of oxalic acid by *B. glumae* is regulated by QS and required to avoid alkaline toxicity (Goo et al., 2012). Production of ammonia in nutrient‐rich medium as a by‐product of the metabolism of amino acids causes alkalinization of the culture medium and toxicity to *B. glumae* (Goo et al., 2012; Nam et al., 2021). Oxalic acid acidifies the culture medium and reverses the alkaline pH toxicity. Acidification of culture medium during bacterial growth is also important to protect acyl‐homoserine lactones (AHLs) from nonenzymatic inactivation, which occurs rapidly at alkaline pH, and thus for QS and virulence (Byers et al., 2002; Le Guillouzer et al., 2020; Yates et al., 2002). To date, no oxalic acid efflux transporter has been identified in *B. glumae*.

These pH changes are probably important during infection as well. Plant pathogens and symbionts replicate in a space outside the plasma membrane of plant cells termed the apoplast (Denny, 1995; Kang et al., 2008), where they must interact with aspects of plant immunity. In the early stages of bacterial infection, plants respond by secreting a number of metabolites resulting in alkalinization of the apoplastic space (Geilfus, 2017; Nachin & Barras, 2000; O'Leary et al., 2016), as well as increased levels of divalent cations including Ca^2+^ and Mg^2+^ (Fones & Preston, 2013; O'Leary et al., 2016). However, little has been reported on the immune responses of rice to infection by *B. glumae*, and the functions of these apoplastic changes in plant defence are poorly understood.

The DedA membrane protein superfamily is found in nearly all living species. DedA proteins may function as membrane transporters (Doerrler et al., 2013; Hama et al., 2022). Our laboratory has characterized DedA proteins in several bacterial species, including *Escherichia coli*, *Burkholderia thailandensis*, and *B. glumae* (Iqbal et al., 2021; Panta et al., 2019). Simultaneous deletion of the *E. coli* DedA family genes *yqjA* and *yghB* (encoding proteins with c.60% amino acid identity) results in altered proton motive force (PMF), cell division defects, induction of envelope stress responses, and sensitivity to elevated temperature, alkaline pH, antibiotics, and biocides (Kumar & Doerrler, 2015; Sikdar et al., 2013; Sikdar & Doerrler, 2010; Thompkins et al., 2008). Our laboratory and others have found that DedA family proteins are required for polymyxin and/or cationic antimicrobial peptide (CAMP) resistance of *Salmonella enterica* (Shi et al., 2004), *Neisseria meningitidis* (Tzeng et al., 2005), *E. coli* (Weatherspoon‐Griffin et al., 2011), *Klebsiella pneumoniae* (Jana et al., 2017), *Enterobacter cloacae* (Huang et al., 2019), *B. thailandensis* (Panta et al., 2019), and *B. glumae* (Iqbal et al., 2021). We named the *Burkholderia* DedA family protein DbcA (DedA of *Burkholderia* required for CAMP resistance). Recent work reported the identification of DedA family proteins in a screen for potential recycling flippases of the lipid undecaprenyl‐phosphate, produced during synthesis of peptidoglycan and through other pathways (Roney & Rudner, 2022; Sit et al., 2022).

*B. glumae* DbcA displays approximately 73% amino acid identity with *B. thailandensis* DbcA. We showed that deletion of *dbcA* causes sensitivity to colistin, decreased toxoflavin production, and loss of virulence (Iqbal et al., 2021). We could replicate these effects on toxin production and loss of virulence with sodium bicarbonate, which dissipates the ΔpH component of the PMF (Farha et al., 2020), and proposed this as a chemical intervention for BPB. In the present study, we investigate whether DbcA is required to maintain proper QS in *B. glumae*. We report that *B. glumae* Δ*dbcA* does not acidify the growth medium due to impaired oxalic acid production. As a result, the culture medium pH of Δ*dbcA* becomes alkaline during the stationary phase and this mutant fails to accumulate AHL and carry out QS signalling. Exposure of *B. glumae* wild type to sodium bicarbonate causes similar effects. These data collectively show that DbcA is required for QS of *B. glumae*.

## 2 RESULTS

### 2.1 *B. glumae* Δ*dbcA* and Δ*obcAB* are unable to acidify the growth medium during the stationary phase

**FIGURE 1:**
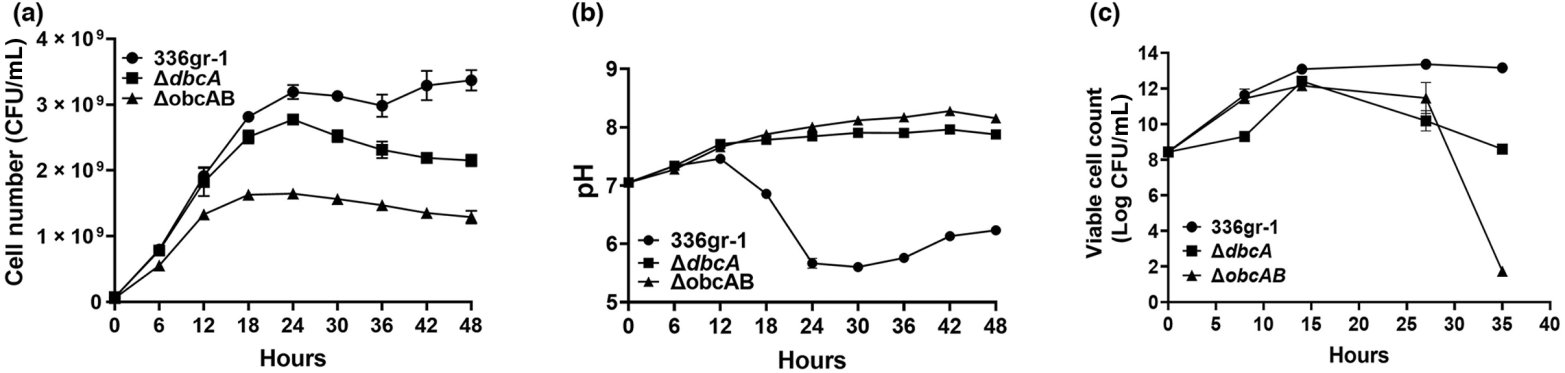
Growth and culture medium pH of *Burkholderia glumae* wild type (336gr‐1), Δ*dbcA*, and Δ*obcAB*. (a) Growth of *B. glumae* strains in LB broth buffered to pH 7.0 with 70 mM Tris measured using a spectrophotometer. Equal numbers of cells (5 × 10^7^) were inoculated into 250‐mL culture flasks containing 40 mL of indicated growth medium and grown at 37°C with shaking. (b) At 6‐h intervals, a portion of the bacterial culture was aseptically removed to measure the medium pH. (c) The viable cell number of *B. glumae*, Δ*dbcA*, and Δ*obcAB*. Aliquots were taken at the indicated time points, serially diluted, and plated on LB agar plates containing 10 μg/mL nitrofurantoin. Colonies were counted after 48 h at 37°C. The data are presented as mean ± standard deviation (*SD*). Each experiment was repeated three times with three independent biological replicates.

The *obcAB* operon is responsible for oxalic acid biosynthesis in *B. glumae* and is needed for acidification of culture medium during the stationary phase (Nakata & He, 2010). We observed that *B. glumae* 336gr‐1 Δ*dbcA* displayed a partial growth defect (Figure 1a) and did not acidify the culture medium at the stationary phase (Figure 1b). We hypothesized that this may be due to a defect in oxalic acid production and secretion. To test this, a *B. glumae* Δ*obcAB* strain was created that synthesizes no oxalic acid.

We measured the growth of *B. glumae* wild type, Δ*dbcA*, and Δ*obcAB* in LB medium buffered with 70 mM Tris at pH 7.0 (Figure 1a) while monitoring the pH of the medium (Figure 1b). We note that while we used LB medium buffered to pH 7.0 with 70 mM bis‐Tris propane (BTP) in our previous study (Iqbal et al., 2021), we found that BTP does not allow wild‐type *B. glumae* to acidify the culture medium, probably due to its wide range of buffering capacity (pH 6.3 to 9.5) (Figure S1). We therefore used LB medium buffered to pH 7.0 with 70 mM Tris for this study. The p*K*_a_ of Tris allows *B. glumae* to produce its natural phenotype (acidification of culture medium) during the stationary phase of growth. During the first 24 h of growth the culture medium pH of wild‐type *B. glumae* decreased from neutral to acidic, while the medium pH of Δ*dbcA* and Δ*obcAB* rose from neutral to alkaline (Figure 1b). *B. glumae* Δ*dbcA* and Δ*obcAB* also showed similar levels of growth and culture medium pH in unbuffered LB medium (Figure S2). Because the cell number shown in Figure 1a was obtained using a spectrophotometer, the growth of all strains was confirmed using plate counts (Figure 1c), which showed that only Δ*obcAB* lost viability at the stationary phase, while Δ*dbcA* maintained viability. We conclude that both DbcA and ObcAB are needed for acidification of the culture medium during growth of *B. glumae*. *B. glumae* Δ*obcAB* was significantly less virulent than the wild type based on an onion scale assay (Iqbal et al., 2021; Figure S3), and expression of a cloned copy of *obcAB* restored the culture medium acidification phenotype of the mutant strain (Figure S4).

### 2.2 *B. glumae* Δ*dbcA* is sensitive to divalent cations and resistance can be restored with external sodium oxalate or acidic pH

**FIGURE 2:**
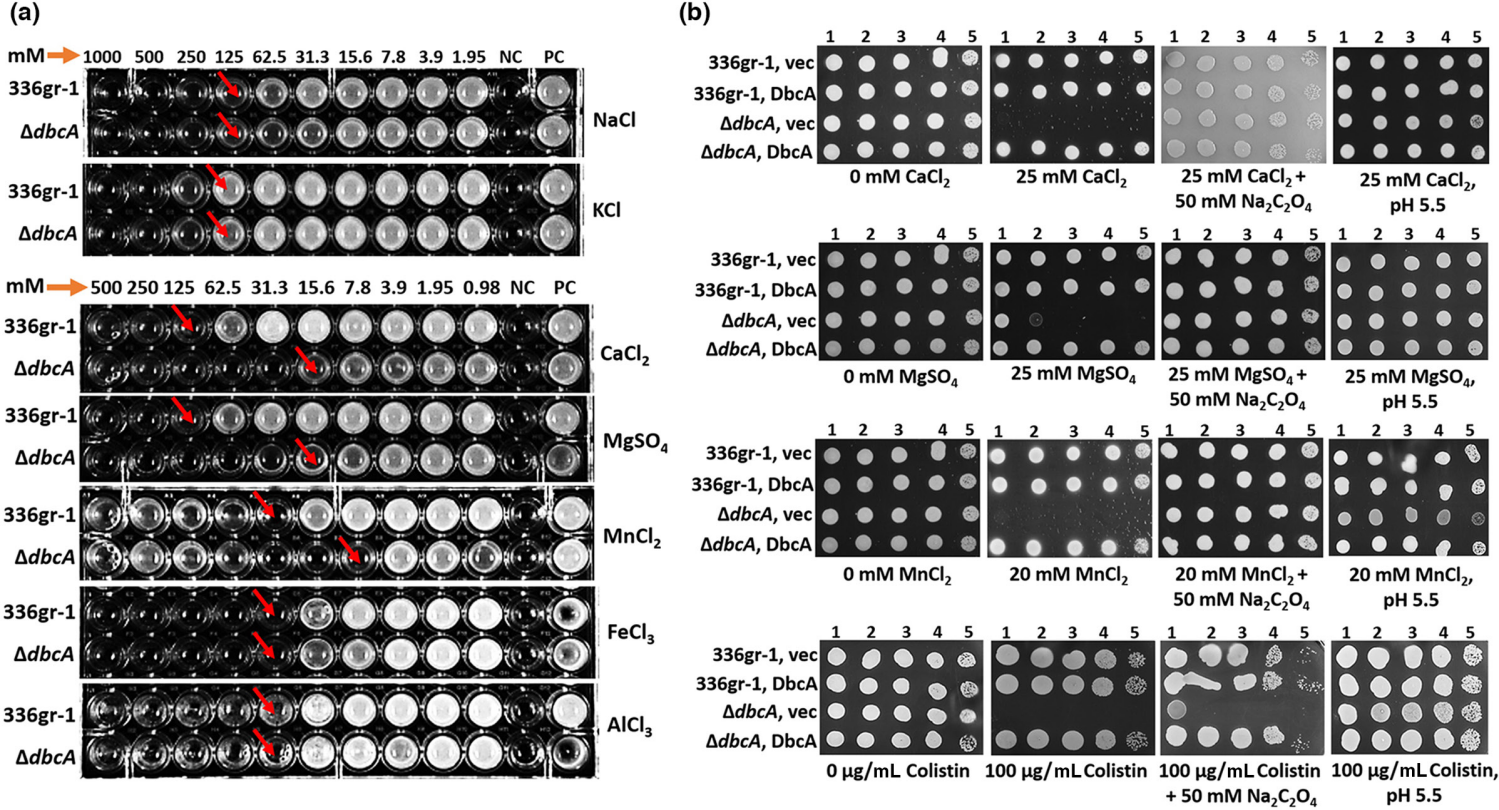
Cation and colistin sensitivity of *Burkholderia glumae* Δ*dbcA*. (a) Minimum inhibitory concentration (MIC) of *B. glumae* wild type (336gr‐1) and Δ*dbcA* in LB broth for sodium chloride (NaCl), potassium chloride (KCl), calcium chloride (CaCl_2_), magnesium sulphate (MgSO_4_), manganese chloride (MnCl_2_), ferric chloride (FeCl_3_), and aluminium chloride (AlCl_3_). The red arrows indicate the approximate MIC. (b) Divalent cation sensitivity on solid medium. Ten‐fold serially diluted cells of *B. glumae* and Δ*dbcA* transformed with control vector (vec) and pSC301 (*dbcA*) were spotted and grown on LB agar containing 100 μg/mL trimethoprim. For determination of cation sensitivity, plates were supplemented with either CaCl_2_, MgSO_4_, or MnCl_2_ at the indicated concentrations. For determination of colistin sensitivity, plates were supplemented with either 0 or 100 μg/mL colistin. Sodium oxalate (Na_2_C_2_O_4_) was added to plates at a concentration of 50 mM to test cation and colistin sensitivity in the presence of external oxalate. LB medium pH was set to 5.5 with hydrochloric acid to test the cation and colistin sensitivity in acidic pH. PC, positive control; NC, negative control. Each experiment was repeated three times with three independent biological replicates. Representative plates are shown.

In a previous study, we showed that *B. thailandensis* Δ*dbcA* is sensitive to the divalent cations Ca^2+^ and Mg^2+^ (Panta & Doerrler, 2021). We were interested in measuring the cation sensitivity of *B*. *glumae* Δ*dbcA* because divalent cations have been reported to be part of the plant immune response to invading bacterial pathogens (Fones & Preston, 2013; O'Leary et al., 2016). We screened *B. glumae* Δ*dbcA* sensitivity against several monovalent (Na^+^ and K^+^), divalent (Ca^2+^, Mg^2+^, and Mn^2+^), and trivalent cations (Al^3+^ and Fe^3+^). We found that *B. glumae* Δ*dbcA* was sensitive only to divalent cations (Figure 2a). The Ca^2+^ and Mg^2+^ minimum inhibitory concentration (MIC) for *B. glumae* Δ*dbcA* was eight times lower than for *B. glumae* wild type, while the Mn^2+^ MIC was four times lower than for *B. glumae* wild type (Figure 2a). The sensitivity of Δ*dbcA* to divalent cations could be reversed by expression of *B. glumae dbcA* from a plasmid (Figure 2b).

It has been shown that oxalic acid is required for aluminium tolerance in *Pseudomonas fluorescens* and transformation of toxic metals in mining sites by the fungus *Beauveria caledonica* (Fomina et al., 2005; Hamel et al., 1999). We tested whether supplementation of external oxalate in LB agar can reverse the divalent cation and colistin sensitivity of *B. glumae* Δ*dbcA*. Oxalic acid is a strong organic acid with p*K*_a1_ 1.25 and p*K*_a2_ 4.27 (Palmieri et al., 2019). To exclude the pH effect, we added oxalate in the form of sodium oxalate, which does not change the pH of the growth medium. We found that 50 mM sodium oxalate completely reversed the divalent cation sensitivity of *B. glumae* Δ*dbcA*, while colistin sensitivity was unchanged (Figure 2b). We found that sensitivity of *B. glumae* Δ*dbcA* to divalent cations and colistin could be reversed by artificially acidifying the medium to pH 5.5 as well (Figure 2b). These results indicate that the divalent cation sensitivity of *B. glumae* Δ*dbcA* is reversed by either addition of external oxalate to the medium or lowering the pH of the growth medium.

### 2.3 *B. glumae* DbcA is required for oxalic acid production

**FIGURE 3:**
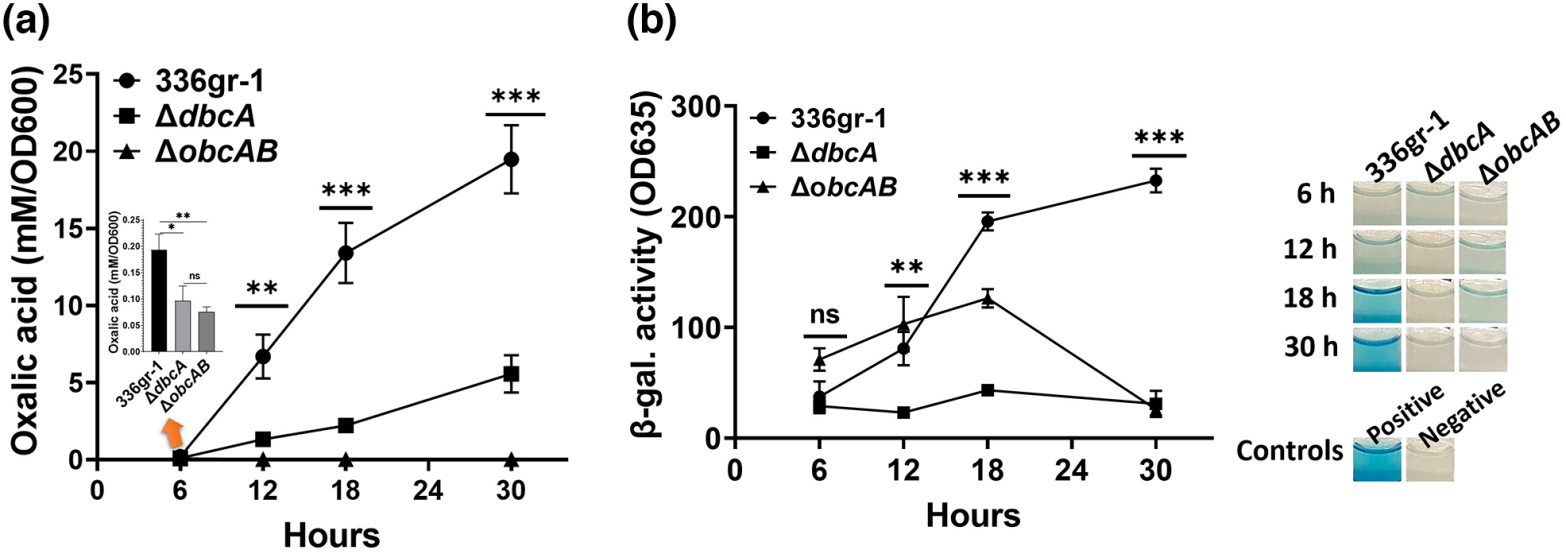
Oxalic acid levels and acyl‐homoserine lactone (AHL) accumulation during growth of *Burkholderia glumae* wild type (336gr‐1), Δ*dbcA*, and Δ*obcAB*. (a) Oxalic acid production in LB broth buffered to 7.0 with 70 mM Tris. Inset bar graph shows oxalic acid levels at 6 h. Equal numbers of cells (5 × 10^7^) were inoculated into either unbuffered or buffered LB broth and grown at 37°C with shaking. Culture supernatants of *B. glumae* strains were collected by centrifugation at the indicated time points and the oxalic acid level was measured. (b) AHL quantification from culture supernatant of indicated strains grown in buffered LB broth based on β‐galactosidase activity. Representative wells are shown. *N*‐octanoyl homoserine (C8‐HSL, 10 μM) was added to the positive control, while no C8‐HSL was added to the negative control. The data are presented as mean ± standard deviation (*SD*). Each experiment was repeated three times with three independent biological replicates. Asterisks indicate a statistically significant difference between *B. glumae* and Δ*dbcA*. *p < 0.05, **p < 0.01, ***p < 0.001; ns, not significant.

*B. glumae* produces ammonia due to metabolism of amino acids in LB culture medium, creating alkaline pH toxicity (Goo et al., 2012; Nam et al., 2021), which is prevented by secretion of oxalic acid (Goo et al., 2012). Because *B. glumae* Δ*dbcA* showed defects in acidification of growth medium during the stationary phase (Figure 1b) and resistance to divalent cations could be restored by addition of external oxalate (Figure 2b), we directly measured oxalic acid production of *B. glumae* strains. We found that wild‐type *B. glumae* consistently produced significantly higher levels of oxalic acid, sufficient to acidify the culture medium, during its growth from the exponential to the stationary phase compared to Δ*dbcA* (Figure 3a). At 6 h, when no difference in growth was observed between the two strains (Figure 1a), *B. glumae* Δ*dbcA* produced significantly lower levels of oxalate compared to the wild type (Figure 3a, inset). The Δ*obcAB* oxalate‐deficient mutant, which was used as a negative control in the experiments, produced no detectable levels of oxalate.

### 2.4 *B. glumae* DbcA is required to preserve AHL molecules at the stationary phase

It has been reported that the QS signalling molecules AHLs undergo inactivation via lactonolysis at alkaline pH (Byers et al., 2002; Le Guillouzer et al., 2020; Yates et al., 2002). Because *B. glumae* Δ*dbcA* cannot acidify growth medium during growth due to a defect in oxalic acid secretion, we asked whether *B. glumae* Δ*dbcA* is deficient in AHL accumulation, which could influence QS‐dependent gene expression. We measured the relative levels of AHLs in growth medium with the β‐galactosidase‐based biosensor strain *Agrobacterium tumefaciens* KYC5. *B. glumae* wild type and Δ*dbcA* produced roughly the same amounts of AHLs at 6 h during the exponential phase (Figure 3b). However, the levels of AHLs dropped dramatically once Δ*dbcA* entered the stationary phase, at 12 to 30 h. *B. glumae* Δ*obcAB* also produced lower levels of AHLs at the stationary phase (Figure 3b). Our results indicate that AHLs of *B. glumae* Δ*dbcA* are probably inactivated due to alkalinization of the growth medium during the stationary phase. Nearly identical results were obtained when oxalic acid and AHL levels were measured from cells grown in unbuffered LB medium (Figure S5).

### 2.5 The *tox* and *obc* operons are down‐regulated in *B. glumae* Δ*dbcA*

**FIGURE 4:**
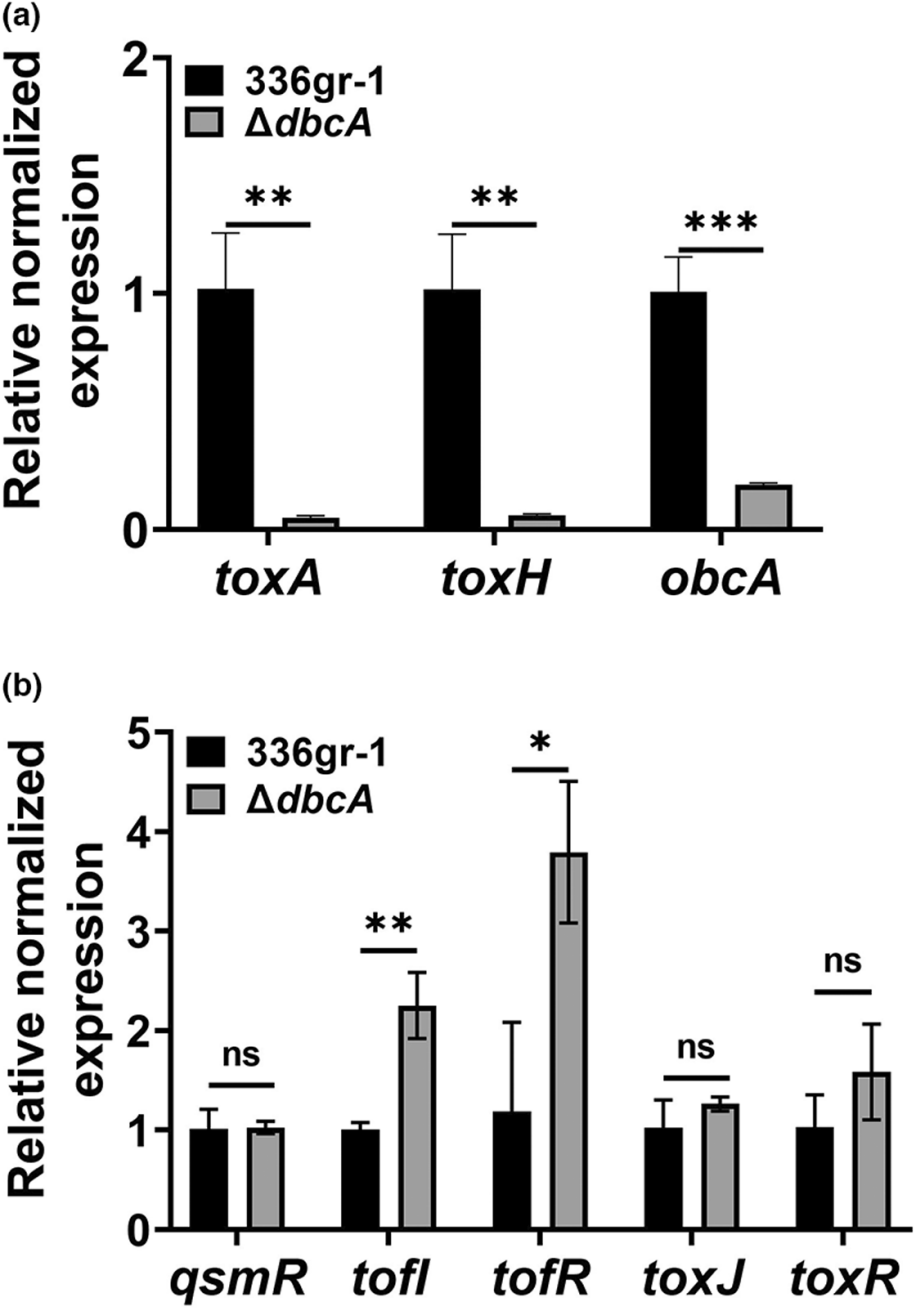
Expression of *toxA*, *toxH*, and *obcA* is down‐regulated in *Burkholderia glumae* Δ*dbcA*. (a) Relative normalized expression levels of *toxA*, *toxH*, and *obcA* in *B. glumae* wild type (336gr‐1) and Δ*dbcA*. (b) Relative normalized expression levels of *qsmR*, *tofI*, *tofR*, *toxJ*, and *toxR* in *B. glumae* and Δ*dbcA*. The data are presented as mean ± standard deviation (*SD*). Each experiment was repeated three times with three independent biological replicates. The statistical significance of differences between *B. glumae* wild type and Δ*dbcA* was calculated using the unpaired Student's *t* test. *p < 0.05, **p < 0.01, ***p < 0.001; ns, not significant.

The *B. glumae toxABCDE* and *toxFGHI* operons are responsible for synthesis and efflux of toxoflavin, respectively (Kim et al., 2004). *B. glumae* regulates the expression of both operons in a QS‐dependent manner mediated by TofI and TofR. Because we found that AHLs of *B. glumae* Δ*dbcA* fail to accumulate during the stationary phase, we measured the expression levels of QS‐regulated genes in *B. glumae* Δ*dbcA*. The expression levels of *toxA*, *toxH*, and *obcA* were determined by reverse transcription‐quantitative PCR (RT‐qPCR) in *B. glumae* wild type and Δ*dbcA*. We found that the expression levels of the *toxA*, *toxH*, and *obcA* genes were significantly reduced in *B. glumae* Δ*dbcA* compared to the wild type (Figure 4a). This result indicates that inactivation of AHLs in alkaline culture medium at least in part affects the expression of both toxoflavin operons in *B. glumae* Δ*dbcA* as well as the oxalic acid operon. However, it remains to be determined how QS regulates oxalic acid production in *B. glumae*.

We then determined if the Δ*dbcA* mutation affects the expression of the QS genes *tofI* and *tofR*, encoding QS signalling proteins, and *qsmR*, *toxJ*, and *toxR*, encoding regulatory proteins. We found that Δ*dbcA* mutation did not affect the expression of *qsmR*, *toxJ*, or *toxR*, but expression of *tofI* and *tofR* was up‐regulated (Figure 4b). This important result suggests that the reduction in AHL levels during the stationary phase is solely due to alkalinization of the medium and is not due to a defect in AHL production, because the mutant strain is able to induce expression of genes involved in AHL synthesis.

### 2.6 Treatment with sodium bicarbonate prevents oxalic acid production

**FIGURE 5:**
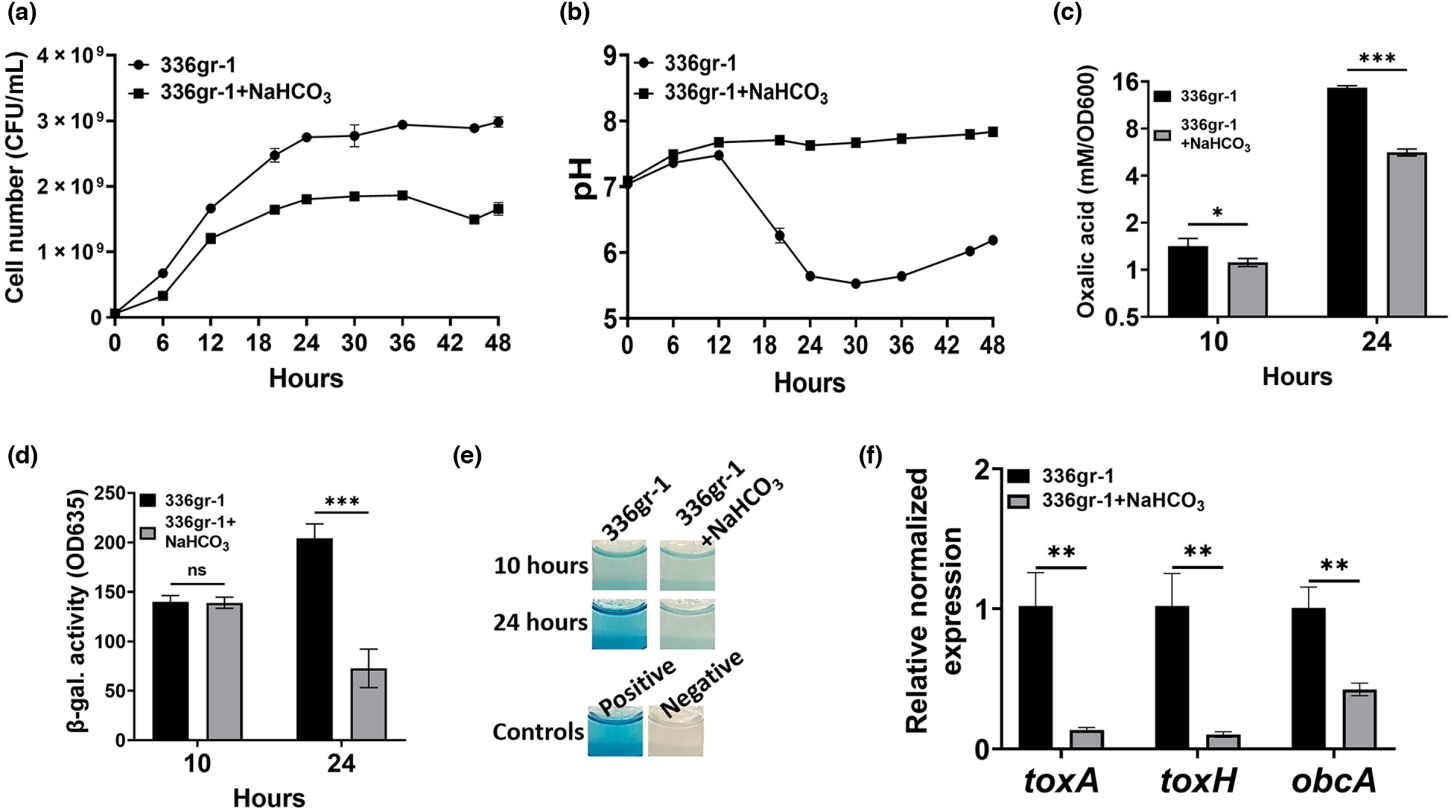
Sodium bicarbonate (NaHCO_3_) reduces oxalic acid production in *Burkholderia glumae*. (a, b) Growth and culture medium pH of *B. glumae* in buffered LB broth with or without 5 mM NaHCO_3_. Equal numbers of cells (5 × 10^7^) were inoculated into culture flasks containing buffered LB medium supplemented with either 0 or 5 mM NaHCO_3_. Bacterial cultures were grown at 37°C with shaking for 48 h. (c) Oxalic acid measurement of *B. glumae* grown in buffered LB broth with or without 5 mM NaHCO_3_. (d) Acyl‐homoserine (AHL) quantification from culture supernatant of *B. glumae* grown in buffered LB broth with or without 5 mM NaHCO_3_ based on β‐galactosidase activity. (e) Representative wells are shown. *N*‐octanoyl homoserine (C8‐HSL, 10 μM) was added to the positive control, while no C8‐HSL was added to the negative control. (f) Expression levels of *toxA*, *toxH*, and *obcA* in *B. glumae* grown in buffered LB broth with or without NaHCO_3_. The data are presented as mean ± standard deviation (*SD*). Each experiment was repeated three times with three independent biological replicates. *p < 0.05, **p < 0.01, ***p < 0.001; ns, not significant.

Previously, we showed that *B. glumae* DbcA is required to maintain normal PMF (Iqbal et al., 2021). Sodium bicarbonate (NaHCO_3_) dissipates the ΔpH component of PMF at physiological concentrations (Farha et al., 2018, 2020; Rose et al., 2020). Therefore, we asked whether treatment of *B. glumae* with 5 mM NaHCO_3_, which does not by itself affect the pH of the culture medium, could reduce oxalic acid production and cause alkalinization of the culture medium. First, we analysed the growth and culture medium pH of *B. glumae* wild type grown with either 0 or 5 mM NaHCO_3_ in LB medium buffered with 70 mM Tris (pH 7.0). We found that *B. glumae* wild type grown with 5 mM NaHCO_3_ did not acidify the growth medium and showed a slight growth defect in the stationary phase compared to the wild‐type *B. glumae* grown without NaHCO_3_ (Figure 5a,b). This pattern is like that observed with Δ*dbcA* when grown in buffered LB medium (Figure 1b).

We measured oxalic acid production in wild‐type *B. glumae* grown with 0 and 5 mM NaHCO_3_. To exclude the effect of cell number on the assay, the oxalic acid production level was measured at the exponential (10 h) and the stationary phase (24 h). We found that wild‐type *B. glumae* grown with 5 mM NaHCO_3_ produced significantly less oxalic acid at 10 h (Figure 5c), and little difference in growth was found (Figure 5a). *B. glumae* wild type grown with NaHCO_3_ also produced significantly less oxalic acid at 24 h compared to the strain grown without NaHCO_3_ (Figure 5c). We then tested the effect of NaHCO_3_ on AHL production during the stationary phase and found that *B. glumae* wild type grown with NaHCO_3_ was compromised for accumulation of AHL at 24 h (Figure 5d,e). However, we did not find a significant difference in AHL levels at 10 h (Figure 5d,e), when the culture medium pH of both strains is near neutral (Figure 5d,e). We also measured the expression levels of *toxA*, *toxH*, and *obcA* for *B. glumae* wild type grown with or without NaHCO_3_. We found that expression of these genes was significantly down‐regulated in wild‐type *B. glumae* grown with NaHCO_3_ (Figure 5f). These results indicate that oxalic acid is probably secreted in a PMF‐dependent manner. Disruption of the PMF with NaHCO_3_ causes alkalinization of the culture medium, inactivation of AHLs, and down‐regulation of virulence genes (*toxA* and *t* *oxH*) and oxalate biosynthesis genes (*obcAB*).

### 2.7 External C8‐HSL rescues oxalic acid production in *B. glumae* Δ*dbcA*

**FIGURE 6:**
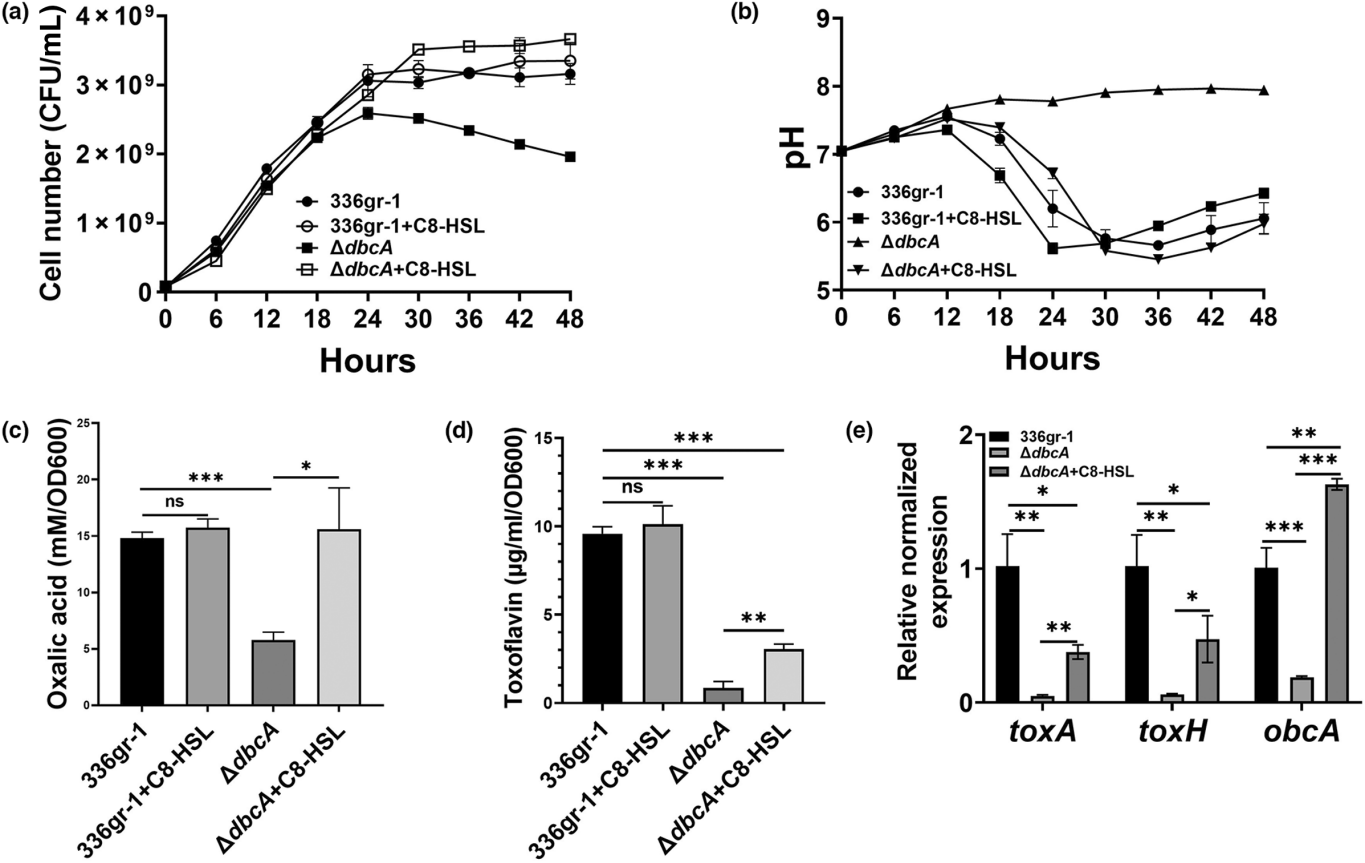
*N*‐octanoyl homoserine (C8‐HSL) restores oxalic acid production in *Burkholderia glumae* Δ*dbcA*. (a, b) Growth and culture medium pH of *B. glumae* wild type (336gr‐1) and Δ*dbcA* in buffered LB broth with or without 5 μM C8‐HSL. Equal numbers of cells (5 × 10^7^) were inoculated in a culture flask containing buffered LB broth and grown at 37°C with shaking. (c) Oxalic acid measurement of *B. glumae* and Δ*dbcA* grown in buffered LB broth with or without C8‐HSL. (d) Toxoflavin production by *B. glumae* and Δ*dbcA* grown in buffered LB broth with or without C8‐HSL. (e) Expression levels of *toxA* and *toxH*, and *obcA* in *B. glumae* and Δ*dbcA* grown in buffered LB broth with or without C8‐HSL. The data is presented as mean ± standard deviation (*SD*). Each experiment was repeated three times with three independent biological replicates. *p < 0.05, **p < 0.01, ***p < 0.001; ns, not significant.

It has been reported that alkalinization of the culture medium in *B. glumae* BGR1 QS mutants, Δ*tofI* and Δ*qsmR*, can be reversed by addition of external C8‐HSL in growth medium (Goo et al., 2012). Because we showed that *B. glumae* Δ*dbcA* was deficient for AHL accumulation during the stationary phase, we tested if addition of external C8‐HSL reverses the culture medium alkalinization. We analysed the growth and culture medium pH of *B. glumae* wild type and Δ*dbcA* grown with or without a physiologically relevant concentration of 5 μM C8‐HSL in buffered LB medium. We found that addition of C8‐HSL completely reversed culture medium alkalinization and that *B. glumae* Δ*dbcA* displayed a growth rate similar to that of the wild type (Figure 6a,b). Addition of C8‐HSL did not affect the growth and culture medium pH of the wild type (Figure 6a,b). We measured oxalic acid production in *B. glumae* wild type and Δ*dbcA* grown with or without C8‐HSL and found that its addition completely restored oxalic acid production in *B. glumae* Δ*dbcA* (Figure 6c). C8‐HSL also provided a partial recovery of toxoflavin production by *B. glumae* Δ*dbcA* (Figure 6d). These data collectively show that a defect in QS is linked to each of these phenotypes of Δ*dbcA* and there may exist additional contributors in the case of toxoflavin production.

We then measured the expression levels of *toxA*, *toxH*, and *obcA* in *B. glumae* wild type and Δ*dbcA* grown with or without C8‐HSL. We found that expression of *obcA* was significantly increased in *B. glumae* Δ*dbcA* grown with C8‐HSL (Figure 6e). This result indicated that external C8‐HSL induces expression of the *obcAB* operon, which in turn produces more oxalic acid to reverse the culture medium alkalinization of *B. glumae* Δ*dbcA* (Figure 6b), consistent with the observation that oxalate production is regulated in a QS‐dependent manner (Goo et al., 2012). We also found that the expression levels of *toxA* and *toxH* were increased in *B. glumae* Δ*dbcA* grown with C8‐HSL, albeit not to the levels observed in wild‐type cells (Figure 6e). This result is consistent with the partial complementation of toxoflavin production in *B. glumae* Δ*dbcA* grown with C8‐HSL (Figure 6d).

## 3 DISCUSSION

Previously, we showed that DbcA is required for colistin resistance, toxoflavin production, and virulence of *B. glumae*. Chemical alteration of the PMF by NaHCO_3_ treatment can also cause loss of virulence of *B. glumae* (Iqbal et al., 2021). We proposed NaHCO_3_ as a potential chemical agent for BPB intervention in rice. In this study, we examined the impact of the Δ*dbcA* mutation and NaHCO_3_ treatment on QS, the master regulator of virulence in *B. glumae* (Chen et al., 2012; Kim et al., 2004, 2007; Lelis et al., 2019; Peng et al., 2020). We show that DbcA is required for oxalic acid production, growth medium acidification at the stationary phase, accumulation of AHL, and transcription of QS‐dependent genes (Figures 1, 3, 4, and 6d). We show that alteration of the PMF in *B. glumae* with NaHCO_3_ can also reduce oxalic acid production and cause alkalinization of the culture medium, which in turn results in reduced AHL levels in the stationary phase (Figure 5). We also show that addition of external C8‐AHL can restore the oxalic acid production and medium acidification phenotype in *B. glumae* Δ*dbcA* (Figure 6). We show for the first time that *B. glumae* DbcA is required for maintenance of proper QS via its necessity for oxalic acid secretion.

*Burkholderia* and other bacterial species use amino acids as a major carbon source in rich LB medium and produce ammonia due to deamination of amino acids (Goo et al., 2012). Production of ammonia increases the pH of the culture medium, causing alkaline pH toxicity to the bacterial cell. *Burkholderia* species produce and secrete oxalic acid to neutralize the ammonia‐mediated alkaline pH toxicity (Goo et al., 2012; Nam et al., 2021). It has been reported that *B. glumae* BGR1 QS (Δ*qsmR* and Δ*tofI*) and oxalate (Δ*obcA* and Δ*obcB*) mutants display a “massive population crash” when the culture medium pH exceeds 8.0 (Goo et al., 2012). We created a *B. glumae* Δ*obcAB* mutant to compare the growth of an oxalate‐deficient mutant with that of Δ*dbcA* and analyse the loss of viability during the stationary phase. We found that *B. glumae* Δ*obcAB* underwent such a population crash during the stationary phase, but this was not observed for Δ*dbcA* although the growth medium of both strains underwent alkalinization to a similar extent. It is possible that the low amount of oxalic acid produced by Δ*dbcA* (Figure 3a) allows better population survival into the stationary phase.

Oxalic acid is a strong metal chelator that can form an oxalate–metal complex (Fomina et al., 2005; Palmieri et al., 2019) and may therefore chelate divalent cations (Ca^2+^, Mg^2+^, and Mn^2+^). As a result, *B. glumae* Δ*dbcA* showed resistance to divalent cations when external oxalate was provided, even when delivered in the form of a sodium salt, suggesting a direct role for oxalate in reducing sensitivity to divalent cations. Among all metals, the role of Ca^2+^ in the innate immune responses of plants is well understood (Fones & Preston, 2013; Gao et al., 2021). The plant apoplast is a dynamic compartment containing water, nutrients, sugars, and organic acids (Sattelmacher, 2001). The apoplast is surrounded by cell walls and can support the growth of pathogenic bacteria (O'Leary et al., 2016). The plant cell wall contains Ca^2+^, which acts as a secondary intracellular messenger (Nishad et al., 2020). The increased concentration of cytosolic Ca^2+^ triggers several pathogen‐mediated immune responses, including accumulation of H_2_O_2_ and generation of oxidative burst at the infection site (Grant et al., 2000). Plants can also alkalize the apoplastic pH in response to pathogen invasion in a response mediated by plant peptide–receptor complexes (Liu et al., 2022). In this context, it is plausible that *B. glumae* may suppress the pathogen‐mediated plant innate immunity by secreting oxalic acid to chelate apoplastic Ca^2+^ and acidify the apoplast.

No oxalic acid efflux transporter has been identified in *B. glumae*. However, the anaerobic gram‐negative bacterium *Oxalobacter formigenes* encodes an oxalate:formate antiporter that imports oxalate in exchange for formate (Hirai & Subramaniam, 2004). A secondary oxalate efflux transporter (FpOAR) has been identified in the fungus *Fomitopsis palustris* (Watanabe et al., 2010). FpOAR is a PMF‐dependent oxalate efflux transporter and displays no similarity with other known oxalate transporters. The export activity of FpOAR can be significantly inhibited by abolishing either ΔΨ or the ΔpH component of the PMF (Watanabe et al., 2010). It is possible that *B. glumae* Δ*dbcA* initially secretes less oxalic acid due to a compromised PMF (Iqbal et al., 2021). Alternatively, DbcA may be directly involved in oxalic acid secretion.

We used NaHCO_3_ to verify that PMF is required for oxalic acid production. Sodium bicarbonate is a common buffer and can dissipate the ΔpH component of the PMF at physiological concentrations and modify bacterial sensitivity to several types of antibiotics (Farha et al., 2018, 2020; Rose et al., 2020). Previously, we showed that NaHCO_3_ alters the PMF by partially increasing ΔΨ in *B. glumae* wild type, chemically replicating the Δ*dbcA* phenotype (Iqbal et al., 2021). We tested whether alteration of the PMF with NaHCO_3_ can reduce oxalic acid production and found a significant reduction (Figure 5c). Due to impaired oxalic acid production, *B. glumae* wild type grown with NaHCO_3_ could not acidify the culture medium and was deficient in AHL production during the stationary phase (Figure 5b,d). This result suggests that alteration of the PMF in *B. glumae* can reduce oxalic acid production, creating alkaline conditions, resulting in degradation of AHLs and down‐regulation of toxoflavin and oxalic acid production. *B. glumae* DbcA plays an important role in maintaining normal oxalic acid production and QS. All these effects are reversed by addition of C8‐HSL to the culture medium (Figure 6).

*B. glumae* regulates its virulence factors in a QS‐dependent manner (Chen et al., 2012; Kim et al., 2004). We tested whether alkalinization of the culture medium can affect QS in *B. glumae* Δ*dbcA*. While *B. glumae* wild type and Δ*dbcA* accumulated similar levels of AHLs in their early phases of growth, *B. glumae* Δ*dbcA* accumulated a significantly lower level of AHLs when the culture medium pH became alkaline in the stationary phase (Figure 3b). Because the stability of AHLs is highly dependent on the pH of the culture medium (Byers et al., 2002; Le Guillouzer et al., 2020; Yates et al., 2002), it is likely that the reduced AHL levels measured during the stationary phase are due to the alkaline pH of the medium, and this in turn is responsible, at least in part, for down‐regulation of the *tox* and *obc* operons in *B. glumae* Δ*dbcA*.

**FIGURE 7:**
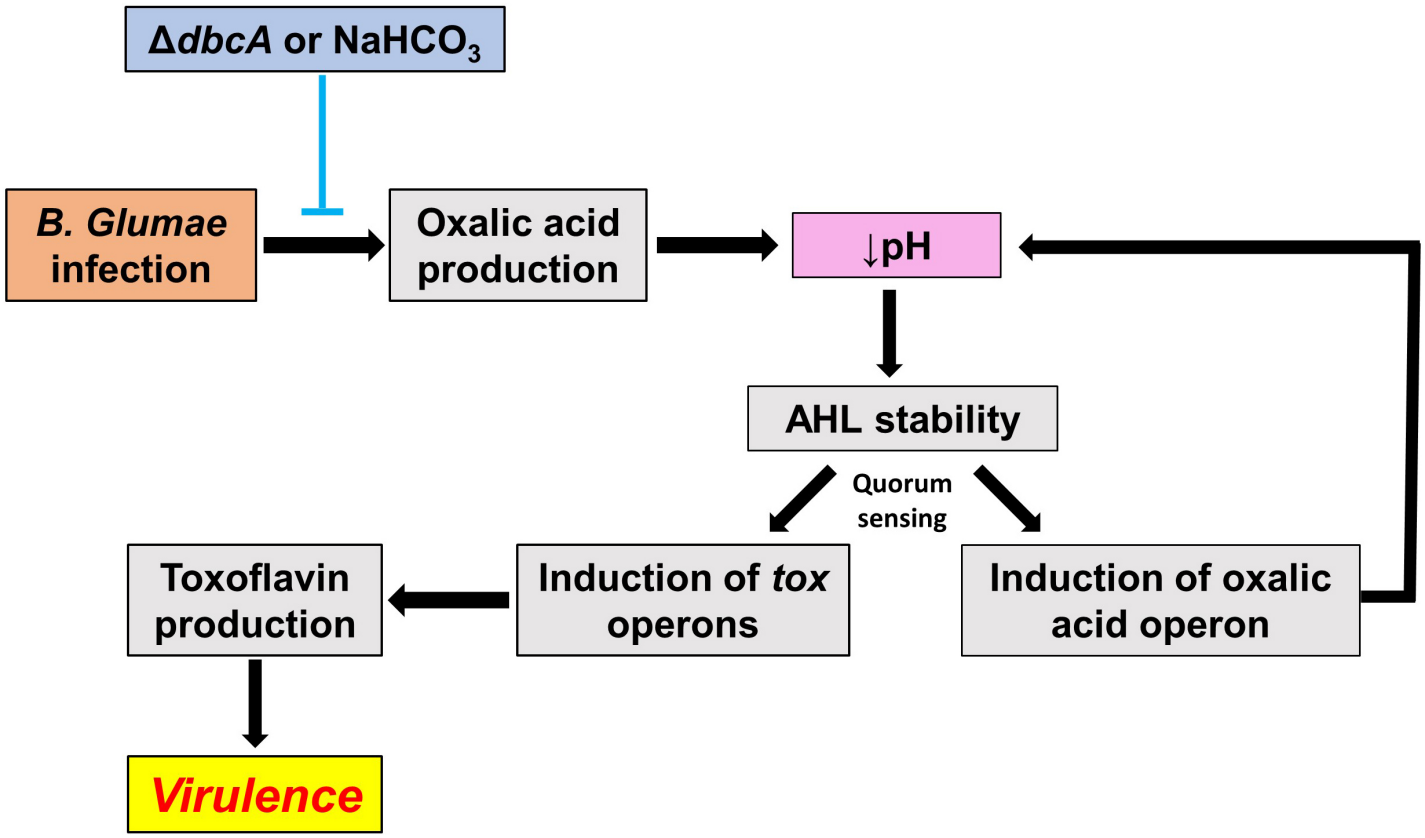
Inactivation of *dbcA* or treatment with NaHCO_3_ results in a series of events leading to loss of toxoflavin production and virulence of *Burkholderia glumae*. Secretion of oxalic acid lowers the pH of the bacterial environment, which prevents nonenzymatic degradation of quorum sensing (QS) AHL signalling molecules (Yates et al., 2002). Plants respond to bacterial infection by producing metabolites that cause alkalinization of the apoplastic space (Geilfus, 2017; Nachin & Barras, 2000; O'Leary et al., 2016). QS activates expression of the *tox* operons required for virulence and the *obc* operon for oxalic acid synthesis (Kim et al., 2004; Nakata & He, 2010). Reduction of oxalic acid secretion by *B. glumae* Δ*dbcA* or exposure to NaHCO_3_ prevents acidification, interfering with QS and *tox* expression, which in turn reduces the virulence of *B. glumae* (Goo et al., 2012). Loss of QS also represses the expression of the *obc* operon (Goo et al., 2017) and further reduces oxalic acid production and potentially amplifies the alkaline pH conditions.

Our results indicate that both DbcA and QS are required for oxalic acid production and growth of *B. glumae*. From our results, we conclude that *B. glumae* DbcA is required to establish a synergistic link between the PMF and QS in which both are presumably dependent upon each other for the regulation of toxoflavin production and virulence (Figure 7). Evidence for a direct role for DbcA in oxalic acid secretion awaits further structural and biochemical studies.

## 4 EXPERIMENTAL PROCEDURES

### 4.1 Growth medium, plasmids, bacterial strains, and chemicals

Table 1 lists bacterial strains and plasmids used in this study. *B. glumae* and *E. coli* were grown in LB medium (1% NaCl, 1% tryptone, 0.5% yeast extract). *E. coli* RHO3 was grown in LB containing 200 μg/mL 2,6‐diaminopimelic acid (DAP; VWR). Some experiments used LB medium buffered with 70 mM Tris (pH 7.0). The AHL biosensor *Agrobacterium tumefaciens* KYC5 was grown in AT medium at 28°C (Tempé et al., 1977). Antibiotics were purchased from either Sigma‐Aldrich or VWR and used at the following concentrations: nitrofurantoin (Nit) 100 μg/mL (for selection of *B. glumae*), kanamycin (Kan) 30 μg/mL (*E. coli*) and 100 μg/mL (*B. glumae*), and trimethoprim (Tmp) 100 μg/mL (*B. glumae* and *E. coli*). Tetracycline (4.5 μg/mL), spectinomycin (50 μg/mL), and gentamycin (100 *μ*g/mL) were used for *A. tumefaciens* KYC5. X‐gal (5‐bromo‐4‐chloro‐3‐indolyl β‐d‐galactopyranoside) was purchased from VWR. Tris base and bis‐Tris propene were purchased from J.T. Baker and Sigma‐Aldrich, respectively. *N*‐octanoyl homoserine lactone (C8‐HSL) and toxoflavin were purchased from Sigma‐Aldrich and Cayman Chemical, respectively. Table S1 lists oligonucleotide primers used in this study (purchased from Sigma‐Aldrich).

**TABLE 1:** Bacterial strains and plasmids used in this study.

Strain/plasmid	Description	Source
*Escherichia coli*		
XL1 Blue	*recA* *1* *endA1 gyrA96 thi‐1 hsdR17 supE44 relA1 lac* F′ *proAB lacI* ^*q*^ *Z*ΔM15 Tn*10* (Tet^R^)	Stratagene
RHO3	SM10 (λ*pir*) Δ*asd*::*FRT ΔaphA*::*FRT*, Kan^S^	López et al. (2009)
S17‐1λ*pir*	λ*pir*, *recA−*	de Lorenzo et al. (1993)
HB101 (pRK2013::Tn*7*)	*recA* ^−^, pRK2013::Tn*7*	Ditta et al. (1980)
*Burkholderia glumae*		
336gr‐1	Wild‐type *B. glumae*	Chen et al. (2012)
Δ*dbcA*:FRT	336gr‐1 Δ*dbcA*:Tmp^S^	Iqbal et al. (2021)
Δ*obcAB* (LSUBP146)	336gr‐1 Δ*obcAB*	This study
*Agrobacterium tumefaciens*		
KYC5 (pJZ372)(pJZ384)(pJZ410)	Ti plasmidless AHLs biosensor strain carrying three plasmids: pJZ372::*traI‐lacZ*, pJZ384::T7‐*traR*, and pJZ410::T7 polymerase. Tet^R^, Spt^R^, Gen^R^	Zhu et al. (2003)
*Plasmids*		
pSCrhaB2	Expression vector; ori_pBBR1_, *rhaR*, *rhaS*, *P*_*rhaB*_, Tmp^R^, mob^+^	Cardona and Valvano (2005)
pSC301	pSCrhaB2 expressing *B. glumae dbcA*	Iqbal et al. (2021)
pSC‐A‐Amp/Kan	pUC *ori*, f1 *ori*, *lacZ′*, A blunt‐ended PCR cloning vector, Kan^R^, Amp^R^	Agilent Technologies
pKKSacB	A gene knockout suicide vector; RP4 *oriT*, R6K *γ‐ori*, *sacB* ^+^, Kan^R^	Chen et al. (2012)
pKKSacBΔ*obcAB*	Deletion construct of the *obcAB* operon cloned into pKKSacB	This study
pSC700	pSCrhaB2 expressing *B. glumae obcAB*	This study

Abbreviations: Amp, ampicillin; Gen, gentamycin; Kan, kanamycin; Spt, spectinomycin; Tet, tetracycline; Tmp, trimethoprim.

### 4.2 Analysis of bacterial growth and culture medium pH

*B. glumae* strains were directly streaked onto LB agar plates containing no antibiotics from −80°C freezer stocks. Plates were incubated for 36 or 48 h. Bacterial colonies were transferred from LB agar plates with sterile loops and suspended in 1 mL sterile LB medium. Equal numbers of cells (5 × 10^7^) were inoculated into 250‐mL conical flasks containing 40 mL fresh medium without antibiotics and grown for up to 48 h at 37°C with shaking. At 6‐h intervals, aliquots of bacterial cultures were aseptically removed from flasks to measure the bacterial cell number using a Bio‐Rad Smartspec Plus spectrophotometer and pH using a standard pH meter.

### 4.3 Transformation and complementation analysis

Heat shock was used for transformation of *E. coli* unless otherwise stated (Froger & Hall, 2007). Biparental conjugation was used for transformation of *B. glumae* as previously described (Iqbal et al., 2021; López et al., 2009). Briefly, recipient *B. glumae* strains were grown in LB medium containing 100 μg/mL Nit at 37°C overnight. Donor *E. coli* RHO3 strains harbouring the Tmp^R^ plasmid were grown in LB medium containing 200 μg/mL DAP and 100 μg/mL Tmp at 37°C overnight. Portions of overnight cultures (0.5 mL each) of recipient and donor strains were washed two times with sterile LB to remove residual antibiotics. The washed cells were mixed, vortexed for 10 s, and spotted on an LB agar plate containing 200 μg/mL DAP. After overnight incubation at 30°C, the mixed cultures were transferred from the LB plate with sterile loops and suspended in 1 mL sterile LB medium. The cultures were washed once with sterile LB medium and resuspended in 1 mL sterile LB. Diluted cultures (1:10 and 1:100) were spread on LB agar plates containing 100 μg/mL Tmp and 50 μg/mL Nit. *B. glumae* colonies harbouring Tmp^R^ plasmid were confirmed by colony PCR (OneTaq Hot Start Quick‐Load 2× Master Mix with GC Buffer, New England Biolabs).

### 4.4 Deletion of the *obcAB* operon from *B. glumae* 336gr‐1

Deletion of the entire *obcAB* operon was performed using homologous recombination as previously described (Melanson et al., 2017). Oligonucleotide primers used for the deletion of *obcAB* are listed in Table S1. The GeneElute Bacterial Genomic DNA Extraction Kit (Sigma‐Aldrich) was used to extract the genomic DNA from *B. glumae* 336gr‐1. Q5 DNA polymerase (New England Biolabs) was used for PCR amplification. The QuickClean 5M PCR purification kit (GeneScript) was used to purify the PCR products.

The 392‐bp upstream and 421‐bp downstream regions of the *obcAB* operon were amplified from *B. glumae* 336gr‐1 genomic DNA. The 3′ end of the upstream fragment has 20 bp homology with the 5′ end of the downstream fragment. The upstream and downstream fragments were assembled by PCR to generate an *obcAB* deletion construct using the Oxalate‐upNEW2FP and Oxalate‐DWN2RP primers (Hilgarth & Lanigan, 2020). The assembled construct (813 bp) was ligated into the PCR cloning vector pSC‐A‐Amp/Kan. The construct was cut from the vector using BamHI and cloned into the knockout vector pKKSacB at the same restriction site to generate pKKSacBΔ*obcAB*. Vector pKKSacBΔ*obcAB* was transformed into *E. coli* S17‐λ*pir* using electroporation and introduced into parental *B. glumae* by triparental conjugation using the *E. coli* HB101 (pRK2013::Tn*7*) helper strain. The transformed *B. glumae* colonies were selected on LB agar medium supplemented with 100 μg/mL Kan and 50 μg/mL Nit. To induce secondary homologous recombination, selected recombinant *B. glumae* colonies were grown in LB at 30°C without antibiotics. Diluted cells (1:100 and 1:1000) from the overnight culture were spread on LB agar medium supplemented with 30% sucrose. The sucrose‐resistant colonies were screened for Kan sensitivity and Nit resistance on LB agar plates. Colonies sensitive to Kan were isolated and genomic DNA was extracted. Deletion of the *obcAB* operon was confirmed by PCR using the Oxalate CompFP and Oxalate CompRP primer set (Figure S6). To construct a complementation plasmid expressing *obcAB*, the operon was PCR‐amplified from genomic DNA of *B. glumae* using primers obc_Fwr_NdeI and obc_Rv_HindIII (Table S1). The purified PCR product was treated with NdeI and HindIII and ligated into the corresponding restriction sites of the expression vector pSCrhaB2, resulting in pSC700 (Table 1).

### 4.5 Susceptibility assays

Sensitivity was measured in liquid medium in 96‐well plates or on solid medium using the broth microdilution method by spotting 5 μL of 10‐fold serially diluted bacterial cells. The plates were incubated at 37°C, and bacterial growth was analysed after 48 h of incubation.

### 4.6 Oxalic acid measurement

Oxalic acid measurement was performed using an oxalate colourimetric assay kit (Abcam) according to the manufacturer's protocol (Liu et al., 2021). Culture supernatants were diluted into 50 μL oxalate assay buffer. Two microlitres of oxalate converter was added and the tubes were incubated at 37°C in the dark. After 1 h, 50 μL of oxalate development master mix (46 μL oxalate development buffer, 2 μL oxalate enzyme mix, and 2 μL oxalate probe) was added to each tube and incubation was continued for 1 h at 37°C in the dark. Absorbance was measured at 450 nm. The oxalic acid concentration was calculated using an oxalate standard curve (Figure S7).

### 4.7 AHL quantification

Production of AHLs was determined using the β‐galactosidase‐based biosensor strain *A. tumefaciens* KYC55 (Zhu et al., 2003), which responds to the presence of AHLs by expressing *lacZ* (β‐galactosidase; Barton et al., 2021; Gupta et al., 2017). Culture supernatants were collected by centrifugation at the indicated time points and passed through a 0.22‐μm filter. An equal number of KYC55 cells (5 × 10^7^ cfu/mL) was added to culture tubes containing 5 mL AT broth supplemented with 40 μg/mL X‐gal. Five microlitres of *B. glumae* supernatant was added and samples were incubated at 28°C for 6 h. Control tubes were supplemented with 0 or 10 μM C8‐HSL. The development of blue colour was read at 635 nm. The absorbance of the negative control was subtracted from the absorbance of each sample. Data were normalized to the OD_600_ value of the tested culture.

### 4.8 Toxoflavin measurement

Measurement of the toxoflavin level in culture media was performed as previously described (Iqbal et al., 2021).

### 4.9 RNA isolation and RT‐qPCR

Overnight cultures were diluted 1:100 in fresh LB medium without antibiotics and grown to an OD_600_ value of approximately 0.6 at 37°C with shaking. Three millilitres of bacterial cultures was collected by centrifugation, washed once with sterile LB, and stored at −80°C overnight. RNA was extracted using the Monarch total RNA mini prep kit (New England Biolabs) according to the manufacturer's instructions. To remove residual genomic DNA, the Turbo DNA‐free kit (Invitrogen) was used to perform a second DNase treatment. The concentration and purity of RNA samples were determined by a NanoDrop instrument (Thermo Scientific).

The Luna Universal One‐Step RT‐qPCR Kit (New England Biolabs) was used to perform qPCR. qPCR was performed in a 20‐μL reaction mixture containing 1× Luna Universal One‐Step reaction mix, 1× Luna WarmStart RT enzyme mix, 0.4 μM gene‐specific forward and reverse primers, and 300 ng RNA. RT‐qPCR was performed using an Applied Biosystems QuantStudio 6 Flex Real‐Time PCR system using SYBR Green I dye with the following PCR conditions: reverse transcription at 55°C for 10 min and initial denaturation at 95°C for 1 min, followed by 40 cycles of denaturation at 95°C for 10 s and extension at 60°C for 1 min. A melt curve was produced for each run at a temperature range from 60°C to 95°C with 1°C increments. The comparative *C*_t_ method (2^−ΔΔ*C*t^) was used to calculate the fold change value of gene expression using the housekeeping gene *gyrA* as an internal reference (Lelis et al., 2019). Statistical analysis was performed using the unpaired Student's *t* test with GraphPad Prism 9.

### 4.10 Statistical analysis

The data are presented in the graphs as mean ± standard deviation (*SD*). Each experiment was repeated three times with three independent biological replicates. Graphs were produced with GraphPad Prism v. 9.0 and statistical significance was calculated using the unpaired Student's *t* test.

## Supporting information


**Figure S1.** Culture medium pH of *Burkholderia glumae* 336gr‐1 in LB broth buffered to pH 7.0 with 70 mM bis‐Tris propane (BTP) with or without 5 mM NaHCO_3_. Equal numbers of cells (5 × 10^7^) were inoculated in 250‐mL culture flasks containing 40 mL of LB broth buffered to pH 7.0 with 70 mM BTP and grown at 37°C with shaking. Culture medium pH was measured at 6‐h intervals.

**Figure S2.** Growth and culture medium pH of *Burkholderia glumae* 336gr‐1, Δ*dbcA*, and Δ*obcAB* strains measured in unbuffered LB broth. Equal numbers of cells (5 × 10^7^) were inoculated in 250‐mL culture flasks containing 40 mL of LB broth and grown at 37°C with shaking. At 6‐h intervals, 3 mL of bacterial cells was removed from the culture flasks to measure the bacterial growth and culture medium pH.

**Figure S3.** Virulence of *Burkholderia glumae* 336gr‐1 and Δ*obcAB* as determined using onion slices. (a) The area of maceration is a measure of the virulence for each strain (Iqbal et al., 2021). Onion slices were infected with 5 × 10^9^ cells of *B. glumae* 336gr‐1 or Δ*obcAB* in 10 μL. As a control, 10 μL of sterile MH2 medium was used. The infected onion slices were incubated at 30°C for 4 days in a humid chamber. (b) Area of maceration (cm^2^) of onion slices produced by *B. glumae* strains. The bar graph shows the results for six replicates. ***p < 0.001.

**Figure S4.** Restoration of culture media acidification by *Burkholderia glumae* Δ*obcAB* through expression of *obcAB*. (a, b) Culture medium pH and growth of *B. glumae* strains. *B. glumae obcAB* was PCR‐amplified from *B. glumae* genomic DNA and the PCR product was ligated into NdeI and HindIII restriction sites of expression vector pSCrhaB2, resulting in pSC700 (Table 1). *B. glumae* 336gr‐1 transformed with control vector pSCrhaB2 (vec) and *B. glumae* Δ*obcAB* transformed with control vector pSCrhaB2 (vec) and pSC700 (*obcAB*) were grown on LB agar containing 50 μg/mL trimethoprim and 0.0005% rhamnose. Equal numbers of cells (5 × 10^7^) were inoculated into 250‐mL culture flasks containing 40 mL of LB broth buffered to 7.0 with 70 mM Tris, 40 μg/mL trimethoprim, and 0.0005% rhamnose and grown at 37°C with shaking for 24 h. After 24 h, 5 mL of bacterial culture was collected to measure pH and cell number. **p < 0.01, ***p < 0.001.

**Figure S5.** Oxalic acid levels and acyl‐homoserine lactone (AHL) accumulation during growth of *Burkholderia glumae* wild type, Δ*dbcA*, and Δ*obcAB* in unbuffered LB broth. (a) Oxalic acid production. The inset bar graph shows oxalic acid levels at 6 h. Equal numbers of cells (5 × 10^7^) were inoculated into 250‐mL culture flasks containing 40 mL of either unbuffered or buffered LB broth and grown at 37°C with shaking. Culture supernatants of *B. glumae* strains were collected by centrifugation at the indicated time points and the oxalic acid level was measured. (b) Acyl‐homoserine (AHL) quantification from culture supernatant of indicated strains. Representative individual wells are shown on the right. *N*‐octanoyl homoserine (C8‐HSL (10 μM) was added to the positive control, while no C8‐HSL was added to the negative control. Asterisks indicate a statistically significant difference between *B. glumae* 336gr‐1 and Δ*dbcA*. *p < 0.05, **p < 0.01, ***p < 0.001.

**Figure S6.** Deletion of the *Burkholderia glumae* 336gr‐1 *obcAB* operon (bglu_2g18780 and bglu_2g18790). (a) The position of the *obcAB* operon in the *B. glumae* 336gr‐1 genome and deletion of the *obcAB* operon from the genome. The *obcAB* operon is located between bglu_2g18770 (LysR family transcriptional regulator) and bglu_2g18800 (NADH‐flavin oxidoreductase). The annealing sites for primers A1F, A1R, A2F, A2R, CompFP, and CompRP are shown. Genes are not drawn to scale. Primers A1F, A1R, A2F, and A2R represent Oxalate‐upNEW2FP, Oxalate‐upNEW2RP, Oxalate‐DWN2FP, and Oxalate‐DWN2RP, respectively. (b) 1% agarose gel stained with ethidium bromide showing the confirmation of deletion of the *obcAB* operon from the *B. glumae* genome. The PCR amplification using CompFP and CompRP primers produced 2325‐ and 254‐bp DNA fragments for parental strain *B. glumae* 336gr‐1 and mutant strain *B. glumae* Δ*obcAB*, respectively. The 1 kb Plus ladder (Life Technologies) was used to determine the sizes of PCR‐amplified DNA fragments.

**Figure S7.** Representative standard curve for oxalic acid determination. The absorbance values of 0, 2, 4, 6, 8, and 10 nmol oxalic acid were imported into Microsoft Excel and a standard curve was generated.

**Table S1.** Oligonucleotide primers used in this study.

## Data Availability

The data that support the findings of this study are available from the corresponding author upon reasonable request.

## References

[mpp13376-bib-0001] Abisado, R.G. , Benomar, S. , Klaus, J.R. , Dandekar, A.A. & Chandler, J.R. (2018) Bacterial quorum sensing and microbial community interactions. mBio, 9, e02331‐17. 29789364 10.1128/mBio.02331-17 PMC5964356

[mpp13376-bib-0002] Barton, I.S. , Eagan, J.L. , Nieves‐Otero, P.A. , Reynolds, I.P. , Platt, T.G. & Fuqua, C. (2021) Co‐dependent and interdigitated: dual quorum sensing systems regulate conjugative transfer of the Ti plasmid and the At megaplasmid in *Agrobacterium tumefaciens* 15955. Frontiers in Microbiology, 11, 605896. 33552018 10.3389/fmicb.2020.605896 PMC7856919

[mpp13376-bib-0003] Byers, J.T. , Lucas, C. , Salmond, G.P. & Welch, M. (2002) Nonenzymatic turnover of an *Erwinia carotovora* quorum‐sensing signaling molecule. Journal of Bacteriology, 184, 1163–1171. 11807077 10.1128/jb.184.4.1163-1171.2002 PMC134803

[mpp13376-bib-0004] Cardona, S.T. & Valvano, M.A.J.P. (2005) An expression vector containing a rhamnose‐inducible promoter provides tightly regulated gene expression in *Burkholderia cenocepacia* . Plamsid, 54, 219–228. 10.1016/j.plasmid.2005.03.004 15925406

[mpp13376-bib-0005] Chen, R. , Barphagha, I.K. , Karki, H.S. & Ham, J.H. (2012) Dissection of quorum‐sensing genes in *Burkholderia glumae* reveals non‐canonical regulation and the new regulatory gene *tofM* for toxoflavin production. PLoS One, 7, e52150. 23284909 10.1371/journal.pone.0052150 PMC3527420

[mpp13376-bib-0006] de Lorenzo, V. , Eltis, L. , Kessler, B. & Timmis, K.N. (1993) Analysis of *Pseudomonas* gene products using *lacI^q^ */*Ptrp*‐*lac* plasmids and transposons that confer conditional phenotypes. Gene, 123, 17–24. 8380783 10.1016/0378-1119(93)90533-9

[mpp13376-bib-0007] Denny, T.P. (1995) Involvement of bacterial polysaccharides in plant pathogenesis. Annual Review of Phytopathology, 33, 173–197. 10.1146/annurev.py.33.090195.001133 18999958

[mpp13376-bib-0008] Ditta, G. , Stanfield, S. , Corbin, D. & Helinski, D.R. (1980) Broad host range DNA cloning system for gram‐negative bacteria: construction of a gene bank of *Rhizobium meliloti* . Proceedings of the National Academy of Sciences of the United States of America, 77, 7347–7351. 7012838 10.1073/pnas.77.12.7347 PMC350500

[mpp13376-bib-0009] Doerrler, W.T. , Sikdar, R. , Kumar, S. & Boughner, L.A. (2013) New functions for the ancient DedA membrane protein family. Journal of Bacteriology, 195, 3–11. 23086209 10.1128/JB.01006-12 PMC3536176

[mpp13376-bib-0010] Farha, M.A. , French, S. , Stokes, J.M. & Brown, E.D. (2018) Bicarbonate alters bacterial susceptibility to antibiotics by targeting the proton motive force. ACS Infectious Diseases, 4, 382–390. 29264917 10.1021/acsinfecdis.7b00194

[mpp13376-bib-0011] Farha, M.A. , MacNair, C.R. , Carfrae, L.A. , El Zahed, S.S. , Ellis, M.J. , Tran, H.R. et al. (2020) Overcoming acquired and native macrolide resistance with bicarbonate. ACS Infectious Diseases, 6, 2709–2718. 32898415 10.1021/acsinfecdis.0c00340

[mpp13376-bib-0012] Fomina, M. , Hillier, S. , Charnock, J. , Melville, K. , Alexander, I.J. & Gadd, G. (2005) Role of oxalic acid overexcretion in transformations of toxic metal minerals by *Beauveria caledonica* . Applied and Environmental Microbiology, 71, 371–381. 15640211 10.1128/AEM.71.1.371-381.2005 PMC544261

[mpp13376-bib-0013] Fones, H. & Preston, G.M. (2013) The impact of transition metals on bacterial plant disease. FEMS Microbiology Reviews, 37, 495–519. 23020129 10.1111/1574-6976.12004

[mpp13376-bib-0014] Froger, A. & Hall, J.E. (2007) Transformation of plasmid DNA into *E. coli* using the heat shock method. Journal of Visualized Experiments, 2007, e253. 10.3791/253 PMC2557105 18997900

[mpp13376-bib-0015] Gadd, G.M. (1999) Fungal production of citric and oxalic acid: importance in metal speciation, physiology and biogeochemical processes. Advances in Microbial Physiology, 41, 47–92. 10500844 10.1016/s0065-2911(08)60165-4

[mpp13376-bib-0016] Gao, M. , He, Y. , Yin, X. , Zhong, X. , Yan, B. , Wu, Y. et al. (2021) Ca^2+^ sensor‐mediated ROS scavenging suppresses rice immunity and is exploited by a fungal effector. Cell, 184, 5391–5404.e17. 34597584 10.1016/j.cell.2021.09.009

[mpp13376-bib-0017] Geilfus, C.M. (2017) The pH of the apoplast: dynamic factor with functional impact under stress. Molecular Plant, 10, 1371–1386. 28987886 10.1016/j.molp.2017.09.018

[mpp13376-bib-0018] Goo, E. , Kang, Y. , Lim, J.Y. , Ham, H. & Hwang, I. (2017) Lethal consequences of overcoming metabolic restrictions imposed on a cooperative bacterial population. mBio, 8, e00042‐17. 28246357 10.1128/mBio.00042-17 PMC5347341

[mpp13376-bib-0019] Goo, E. , Majerczyk, C.D. , An, J.H. , Chandler, J.R. , Seo, Y.S. , Ham, H. et al. (2012) Bacterial quorum sensing, cooperativity, and anticipation of stationary‐phase stress. Proceedings of the National Academy of Sciences of the United States of America, 109, 19775–19780. 23150539 10.1073/pnas.1218092109 PMC3511722

[mpp13376-bib-0020] Grant, M. , Brown, I. , Adams, S. , Knight, M. , Ainslie, A. & Mansfield, J. (2000) The RPM1 plant disease resistance gene facilitates a rapid and sustained increase in cytosolic calcium that is necessary for the oxidative burst and hypersensitive cell death. The Plant Journal, 23, 441–450. 10972870 10.1046/j.1365-313x.2000.00804.x

[mpp13376-bib-0021] Gupta, A. , Bedre, R. , Thapa, S.S. , Sabrin, A. , Wang, G. , Dassanayake, M. et al. (2017) Global awakening of cryptic biosynthetic gene clusters in *Burkholderia thailandensis* . ACS Chemical Biology, 12, 3012–3021. 29087175 10.1021/acschembio.7b00681 PMC5732026

[mpp13376-bib-0022] Ham, J.H. , Melanson, R.A. & Rush, M.C. (2011) *Burkholderia glumae*: next major pathogen of rice? Molecular Plant Pathology, 12, 329–339. 21453428 10.1111/j.1364-3703.2010.00676.x PMC6640401

[mpp13376-bib-0023] Hama, Y. , Morishita, H. & Mizushima, N. (2022) Regulation of ER‐derived membrane dynamics by the DedA domain‐containing proteins VMP1 and TMEM41B. EMBO Reports, 23, e53894. 35044051 10.15252/embr.202153894 PMC8811646

[mpp13376-bib-0024] Hamel, R. , Levasseur, R. & Appanna, V.D. (1999) Oxalic acid production and aluminum tolerance in *Pseudomonas fluorescens* . Journal of Inorganic Biochemistry, 76, 99–104. 10612061 10.1016/s0162-0134(99)00120-8

[mpp13376-bib-0025] Helman, Y. & Chernin, L. (2015) Silencing the mob: disrupting quorum sensing as a means to fight plant disease. Molecular Plant Pathology, 16, 316–329. 25113857 10.1111/mpp.12180 PMC6638422

[mpp13376-bib-0026] Hilgarth, R.S. & Lanigan, T.M. (2020) Optimization of overlap extension PCR for efficient transgene construction. MethodsX, 7, 100759. 32021819 10.1016/j.mex.2019.12.001 PMC6992990

[mpp13376-bib-0027] Hirai, T. & Subramaniam, S. (2004) Structure and transport mechanism of the bacterial oxalate transporter OxlT. Biophysical Journal, 87, 3600–3607. 15339805 10.1529/biophysj.104.049320 PMC1304825

[mpp13376-bib-0028] Huang, L. , Feng, Y. & Zong, Z. (2019) Heterogeneous resistance to colistin in *Enterobacter cloacae* complex due to a new small transmembrane protein. Journal of Antimicrobial Chemotherapy, 74, 2551–2558. 31169899 10.1093/jac/dkz236

[mpp13376-bib-0029] Iqbal, A. , Panta, P.R. , Ontoy, J. , Bruno, J. , Ham, J.H. & Doerrler, W.T. (2021) Chemical or genetic alteration of proton motive force results in loss of virulence of *Burkholderia glumae*, the cause of rice bacterial panicle blight. Applied and Environmental Microbiology, 87, e0091521. 34260305 10.1128/AEM.00915-21 PMC8388836

[mpp13376-bib-0030] Jana, B. , Cain, A.K. , Doerrler, W.T. , Boinett, C.J. , Fookes, M.C. , Parkhill, J. et al. (2017) The secondary resistome of multidrug‐resistant *Klebsiella pneumoniae* . Scientific Reports, 7, 42483. 28198411 10.1038/srep42483 PMC5309761

[mpp13376-bib-0031] Kang, Y. , Kim, J. , Kim, S. , Kim, H. , Lim, J.Y. , Kim, M. et al. (2008) Proteomic analysis of the proteins regulated by HrpB from the plant pathogenic bacterium *Burkholderia glumae* . Proteomics, 8, 106–121. 18050277 10.1002/pmic.200700244

[mpp13376-bib-0032] Kim, J. , Kang, Y. , Choi, O. , Jeong, Y. , Jeong, J.E. , Lim, J.Y. et al. (2007) Regulation of polar flagellum genes is mediated by quorum sensing and FlhDC in *Burkholderia glumae* . Molecular Microbiology, 64, 165–179. 17376080 10.1111/j.1365-2958.2007.05646.x

[mpp13376-bib-0033] Kim, J. , Kim, J.G. , Kang, Y. , Jang, J.Y. , Jog, G.J. , Lim, J.Y. et al. (2004) Quorum sensing and the LysR‐type transcriptional activator ToxR regulate toxoflavin biosynthesis and transport in *Burkholderia glumae* . Molecular Microbiology, 54, 921–934. 15522077 10.1111/j.1365-2958.2004.04338.x

[mpp13376-bib-0034] Kumar, S. & Doerrler, W.T. (2015) *Escherichia coli* YqjA, a member of the conserved DedA/Tvp38 membrane protein family, is a putative osmosensing transporter required for growth at alkaline pH. Journal of Bacteriology, 197, 2292–2300. 25917916 10.1128/JB.00175-15 PMC4524195

[mpp13376-bib-0035] Latuasan, H. & Berends, W. (1961) On the origin of the toxicity of toxoflavin. Biochimica et Biophysica Acta, 52, 502–508. 14462713 10.1016/0006-3002(61)90408-5

[mpp13376-bib-0036] Le Guillouzer, S. , Groleau, M.C. , Mauffrey, F. & Deziel, E. (2020) ScmR, a global regulator of gene expression, quorum sensing, pH homeostasis, and virulence in *Burkholderia thailandensis* . Journal of Bacteriology, 202, e00776‐19. 32312745 10.1128/JB.00776-19 PMC7283594

[mpp13376-bib-0037] Lelis, T. , Peng, J. , Barphagha, I. , Chen, R. & Ham, J.H. (2019) The virulence function and regulation of the metalloprotease gene *prtA* in the plant‐pathogenic bacterium *Burkholderia glumae* . Molecular Plant‐Microbe Interactions, 32, 841–852. 30694091 10.1094/MPMI-11-18-0312-R

[mpp13376-bib-0038] Liu, L. , Song, W. , Huang, S. , Jiang, K. , Moriwaki, Y. , Wang, Y. et al. (2022) Extracellular pH sensing by plant cell‐surface peptide‐receptor complexes. Cell, 185, 3341–3355.e13. 35998629 10.1016/j.cell.2022.07.012

[mpp13376-bib-0039] Liu, M. , Devlin, J.C. , Hu, J. , Volkova, A. , Battaglia, T.W. , Ho, M. et al. (2021) Microbial genetic and transcriptional contributions to oxalate degradation by the gut microbiota in health and disease. eLife, 10, e63642. 33769280 10.7554/eLife.63642 PMC8062136

[mpp13376-bib-0040] López, C.M. , Rholl, D.A. , Trunck, L.A. & Schweizer, H.P. (2009) Versatile dual‐technology system for markerless allele replacement in *Burkholderia pseudomallei* . Journal of Applied and Environmental Microbiology, 75, 6496–6503. 19700544 10.1128/AEM.01669-09 PMC2765137

[mpp13376-bib-0041] Melanson, R.A. , Barphagha, I. , Osti, S. , Lelis, T.P. , Karki, H.S. , Chen, R. et al. (2017) Identification of new regulatory genes involved in the pathogenic functions of the rice‐pathogenic bacterium *Burkholderia glumae* . Microbiology, 163, 266–279. 28036242 10.1099/mic.0.000419

[mpp13376-bib-0042] Munir, E. , Yoon, J.J. , Tokimatsu, T. , Hattori, T. & Shimada, M. (2001) A physiological role for oxalic acid biosynthesis in the wood‐rotting basidiomycete *Fomitopsis palustris* . Proceedings of the National Academy of Sciences of the United States of America, 98, 11126–11130. 11553780 10.1073/pnas.191389598 PMC58694

[mpp13376-bib-0043] Nachin, L. & Barras, F. (2000) External pH: an environmental signal that helps to rationalize pel gene duplication in *Erwinia chrysanthemi* . Molecular Plant‐Microbe Interactions, 13, 882–886. 10939260 10.1094/MPMI.2000.13.8.882

[mpp13376-bib-0044] Nakata, P.A. (2011) The oxalic acid biosynthetic activity of *Burkholderia mallei* is encoded by a single locus. Microbiological Research, 166, 531–538. 21242070 10.1016/j.micres.2010.11.002

[mpp13376-bib-0045] Nakata, P.A. & He, C. (2010) Oxalic acid biosynthesis is encoded by an operon in *Burkholderia glumae* . FEMS Microbiology Letters, 304, 177–182. 20141533 10.1111/j.1574-6968.2010.01895.x

[mpp13376-bib-0046] Nam, Y. , Goo, E. , Kang, Y. & Hwang, I. (2021) Membrane depolarization and apoptosis‐like cell death in an alkaline environment in the rice pathogen *Burkholderia glumae* . Frontiers in Microbiology, 12, 755596. 34712216 10.3389/fmicb.2021.755596 PMC8546246

[mpp13376-bib-0047] Naughton, L.M. , An, S.Q. , Hwang, I. , Chou, S.H. , He, Y.Q. , Tang, J.L. et al. (2016) Functional and genomic insights into the pathogenesis of *Burkholderia* species to rice. Environmental Microbiology, 18, 780–790. 26690879 10.1111/1462-2920.13189

[mpp13376-bib-0048] Nishad, R. , Ahmed, T. , Rahman, V.J. & Kareem, A. (2020) Modulation of plant defense system in response to microbial interactions. Frontiers in Microbiology, 11, 1298. 32719660 10.3389/fmicb.2020.01298 PMC7350780

[mpp13376-bib-0049] O'Leary, B.M. , Neale, H.C. , Geilfus, C.M. , Jackson, R.W. , Arnold, D.L. & Preston, G.M. (2016) Early changes in apoplast composition associated with defence and disease in interactions between *Phaseolus vulgaris* and the halo blight pathogen *Pseudomonas syringae* pv. *phaseolicola* . Plant Cell and Environment, 39, 2172–2184. 10.1111/pce.12770 PMC5026161 27239727

[mpp13376-bib-0050] Ortega, L. & Rojas, C.M. (2021) Bacterial panicle blight and *Burkholderia glumae*: from pathogen biology to disease control. Phytopathology, 111, 772–778. 33206007 10.1094/PHYTO-09-20-0401-RVW

[mpp13376-bib-0051] Palmieri, F. , Estoppey, A. , House, G.L. , Lohberger, A. , Bindschedler, S. , Chain, P.S. et al. (2019) Oxalic acid, a molecule at the crossroads of bacterial–fungal interactions. Advances in Applied Microbiology, 106, 49–77. 30798804 10.1016/bs.aambs.2018.10.001

[mpp13376-bib-0052] Panta, P.R. & Doerrler, W.T. (2021) A *Burkholderia thailandensis* DedA family membrane protein is required for proton motive force dependent lipid a modification. Frontiers in Microbiology, 11, 618389. 33510730 10.3389/fmicb.2020.618389 PMC7835334

[mpp13376-bib-0053] Panta, P.R. , Kumar, S. , Stafford, C.F. , Billiot, C.E. , Douglass, M.V. , Herrera, C.M. et al. (2019) A DedA family membrane protein is required for *Burkholderia thailandensis* colistin resistance. Frontiers in Microbiology, 10, 2532. 31827463 10.3389/fmicb.2019.02532 PMC6849406

[mpp13376-bib-0054] Papenfort, K. & Bassler, B.L. (2016) Quorum sensing signal‐response systems in gram‐negative bacteria. Nature Reviews Microbiology, 14, 576–588. 27510864 10.1038/nrmicro.2016.89 PMC5056591

[mpp13376-bib-0055] Park, J. , Lee, H.‐H. , Jung, H. & Seo, Y.‐S. (2019) Transcriptome analysis to understand the effects of the toxoflavin and tropolone produced by phytopathogenic *Burkholderia* on *Escherichia coli* . Journal of Microbiology, 57, 781–794. 31452043 10.1007/s12275-019-9330-1

[mpp13376-bib-0056] Peng, J. , Lelis, T. , Chen, R. , Barphagha, I. , Osti, S. & Ham, J.H. (2020) *tepR* encoding a bacterial enhancer‐binding protein orchestrates the virulence and interspecies competition of *Burkholderia glumae* through *qsmR* and a type VI secretion system. Molecular Plant Pathology, 21, 1042–1054. 32608174 10.1111/mpp.12947 PMC7368122

[mpp13376-bib-0057] Rattray, J.B. , Thomas, S.A. , Wang, Y. , Molotkova, E. , Gurney, J. , Varga, J.J. et al. (2022) Bacterial quorum sensing allows graded and bimodal cellular responses to variations in population density. mBio, 13, e0074522. 35583321 10.1128/mbio.00745-22 PMC9239169

[mpp13376-bib-0058] Roney, I.J. & Rudner, D.Z. (2022) Two broadly conserved families of polyprenyl‐phosphate transporters. Nature, 613, 729–734. 36450357 10.1038/s41586-022-05587-z PMC10184681

[mpp13376-bib-0059] Rose, W.E. , Bienvenida, A.M. , Xiong, Y.Q. , Chambers, H.F. , Bayer, A.S. & Ersoy, S.C. (2020) Ability of bicarbonate supplementation to sensitize selected methicillin‐resistant *Staphylococcus aureus* strains to β‐lactam antibiotics in an *ex vivo* simulated endocardial vegetation model. Antimicrobial Agents and Chemotherapy, 64, e02072‐19. 31844004 10.1128/AAC.02072-19 PMC7038310

[mpp13376-bib-0060] Sattelmacher, B. (2001) The apoplast and its significance for plant mineral nutrition. New Phytologist, 149, 167–192. 33874640 10.1046/j.1469-8137.2001.00034.x

[mpp13376-bib-0061] Shew, A.M. , Durand‐Morat, A. , Nalley, L.L. , Zhou, X.‐G. , Rojas, C. & Thoma, G. (2019) Warming increases bacterial panicle blight (*Burkholderia glumae*) occurrences and impacts on USA rice production. PLoS One, 14, e0219199. 31295286 10.1371/journal.pone.0219199 PMC6623956

[mpp13376-bib-0062] Shi, Y. , Cromie, M.J. , Hsu, F.F. , Turk, J. & Groisman, E.A. (2004) PhoP‐regulated *salmonella* resistance to the antimicrobial peptides magainin 2 and polymyxin B. Molecular Microbiology, 53, 229–241. 15225317 10.1111/j.1365-2958.2004.04107.x

[mpp13376-bib-0063] Sikdar, R. & Doerrler, W.T. (2010) Inefficient Tat‐dependent export of periplasmic amidases in an *Escherichia coli* strain with mutations in two DedA family genes. Journal of Bacteriology, 192, 807–818. 19880597 10.1128/JB.00716-09 PMC2812453

[mpp13376-bib-0064] Sikdar, R. , Simmons, A.R. & Doerrler, W.T. (2013) Multiple envelope stress response pathways are activated in an *Escherichia coli* strain with mutations in two members of the DedA membrane protein family. Journal of Bacteriology, 195, 12–24. 23042993 10.1128/JB.00762-12 PMC3536178

[mpp13376-bib-0065] Sit, B. , Srisuknimit, V. , Bueno, E. , Zingl, F.G. , Hullahalli, K. , Cava, F. et al. (2022) Undecaprenyl phosphate translocases confer conditional microbial fitness. Nature, 613, 721–728. 36450355 10.1038/s41586-022-05569-1 PMC9876793

[mpp13376-bib-0066] Suzuki, F. , Sawada, H. , Azegami, K. & Tsuchiya, K. (2004) Molecular characterization of the tox operon involved in toxoflavin biosynthesis of *Burkholderia glumae* . Journal of General Plant Pathology, 70, 97–107.

[mpp13376-bib-0067] Tempé, J. , Petit, A. , Holsters, M. , Van Montagu, M. & Schell, J. (1977) Thermosensitive step associated with transfer of the Ti plasmid during conjugation: possible relation to transformation in crown gall. Proceedings of the National Academy of Sciences of the United States of America, 74, 2848–2849. 16592419 10.1073/pnas.74.7.2848 PMC431316

[mpp13376-bib-0068] Thompkins, K. , Chattopadhyay, B. , Xiao, Y. , Henk, M.C. & Doerrler, W.T. (2008) Temperature sensitivity and cell division defects in an *Escherichia coli* strain with mutations in *yghB* and *yqjA*, encoding related and conserved inner membrane proteins. Journal of Bacteriology, 190, 4489–4500. 18456815 10.1128/JB.00414-08 PMC2446817

[mpp13376-bib-0069] Tzeng, Y.L. , Ambrose, K.D. , Zughaier, S. , Zhou, X. , Miller, Y.K. , Shafer, W.M. et al. (2005) Cationic antimicrobial peptide resistance in *Neisseria meningitidis* . Journal of Bacteriology, 187, 5387–5396. 16030233 10.1128/JB.187.15.5387-5396.2005 PMC1196002

[mpp13376-bib-0070] Watanabe, T. , Shitan, N. , Suzuki, S. , Umezawa, T. , Shimada, M. , Yazaki, K. et al. (2010) Oxalate efflux transporter from the brown rot fungus *Fomitopsis palustris* . Applied and Environmental Microbiology, 76, 7683–7690. 20889782 10.1128/AEM.00829-10 PMC2988596

[mpp13376-bib-0071] Weatherspoon‐Griffin, N. , Zhao, G. , Kong, W. , Kong, Y. , Morigen , Andrews‐Polymenis, H. et al. (2011) The CpxR/CpxA two‐component system up‐regulates two Tat‐dependent peptidoglycan amidases to confer bacterial resistance to antimicrobial peptide. Journal of Biological Chemistry, 286, 5529–5539. 21149452 10.1074/jbc.M110.200352 PMC3037666

[mpp13376-bib-0072] White, M. , Heros, E. , Graterol, E. , Chirinda, N. & Pittelkow, C.M. (2020) Balancing economic and environmental performance for small‐scale rice farmers in Peru. Frontiers in Sustainable Food Systems, 4, 564418.

[mpp13376-bib-0073] Yates, E.A. , Philipp, B. , Buckley, C. , Atkinson, S. , Chhabra, S.R. , Sockett, R.E. et al. (2002) *N*‐acylhomoserine lactones undergo lactonolysis in a pH‐, temperature‐, and acyl chain length‐dependent manner during growth of *Yersinia pseudotuberculosis* and *Pseudomonas aeruginosa* . Infection and Immunity, 70, 5635–5646. 12228292 10.1128/IAI.70.10.5635-5646.2002 PMC128322

[mpp13376-bib-0074] Zhu, J. , Chai, Y. , Zhong, Z. , Li, S. & Winans, S.C. (2003) *Agrobacterium* bioassay strain for ultrasensitive detection of *N*‐acylhomoserine lactone‐type quorum‐sensing molecules: detection of autoinducers in *Mesorhizobium huakuii* . Journal of Applied and Environmental Microbiology, 69, 6949–6953. 14602662 10.1128/AEM.69.11.6949-6953.2003 PMC262303

