# Supplementary material for: A membrane protein of the rice pathogen Burkholderia glumae required for oxalic acid secretion and quorum sensing
Source: Mol Plant Pathol. 2023 Jul 10;24(11):1400–13. doi: 10.1111/mpp.13376 (PMC10576180; doi:10.1111/mpp.13376)
Supplement: Supplementary file 1 — Figure S1. Culture medium pH of Burkholderia glumae 336gr‐1 in LB broth buffered to pH 7.0 with 70 mM bis‐Tris propane (BTP) with or without 5 mM NaHCO3. Equal numbers of cells (5 × 10^7^) were inoculated in 250‐mL culture flasks containing 40 mL of LB broth buffered to pH 7.0 with 70 mM BTP and grown at 37°C with shaking. Culture medium pH was measured at 6‐h intervals. [file MPP-24-1400-s008.docx]

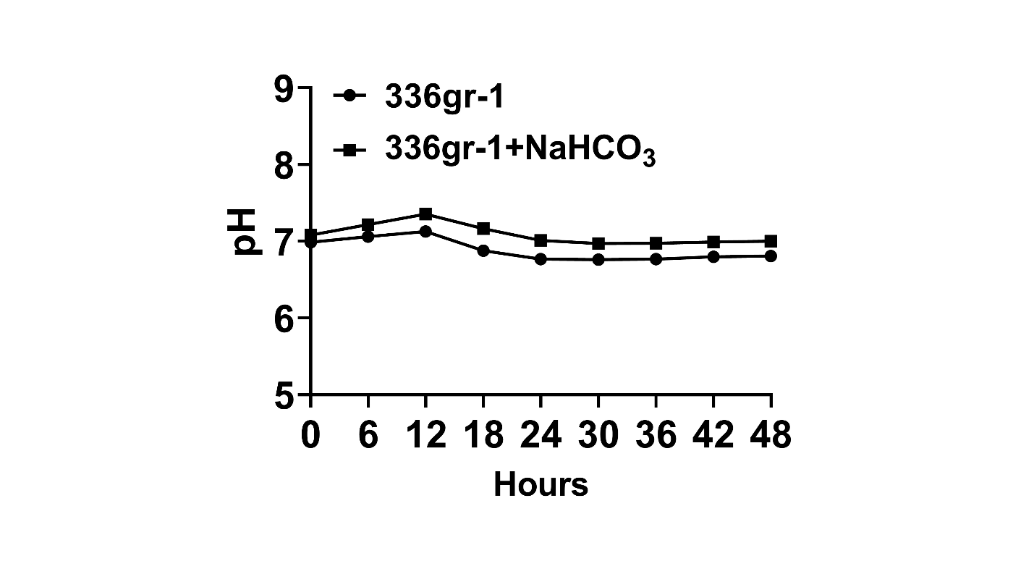


**Figure S1. Culture medium pH of *B. glumae* 336gr-1 in LB broth buffered to pH 7.0 with 70 mM BTP with or without 5 mM NaHCO_3_.** Equal numbers of cells (5 x 10^7^) were inoculated in 250 ml culture flask containing 40 ml of LB broth buffered to pH 7.0 with 70 mM BTP and grown at 37°C with shaking. Culture medium pH was measured at 6-hour intervals.
