# Supplementary material for: A membrane protein of the rice pathogen Burkholderia glumae required for oxalic acid secretion and quorum sensing
Source: Mol Plant Pathol. 2023 Jul 10;24(11):1400–13. doi: 10.1111/mpp.13376 (PMC10576180; doi:10.1111/mpp.13376)
Supplement: Supplementary file 2 — Figure S2. Growth and culture medium pH of Burkholderia glumae 336gr‐1, ΔdbcA, and ΔobcAB strains measured in unbuffered LB broth. Equal numbers of cells (5 × 10^7^) were inoculated in 250‐mL culture flasks containing 40 mL of LB broth and grown at 37°C with shaking. At 6‐h intervals, 3 mL of bacterial cells was removed from the culture flasks to measure the bacterial growth and culture medium pH. [file MPP-24-1400-s007.docx]

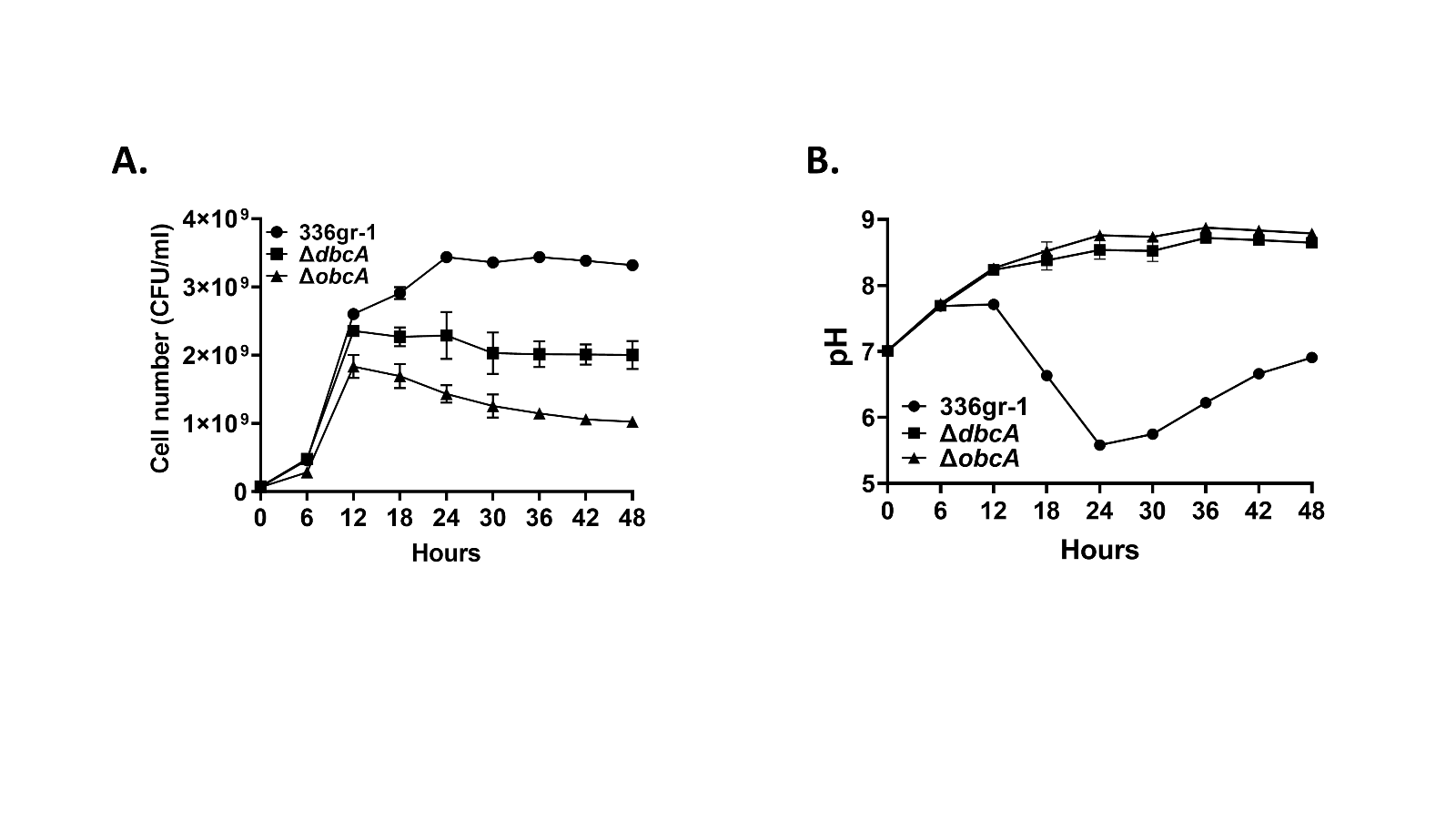


**Figure S2. Growth and culture medium pH of *B. glumae* 336gr-1, Δ*dbcA*, and Δ*obcAB* strains measured in unbuffered LB broth**. An equal number of cells (5 x 10^7^) were inoculated in 250 ml culture flask containing 40 ml of LB broth and grown at 37°C with shaking. At 6-hour intervals, three ml of bacterial cells was removed from culture flask to measure the bacterial growth and culture medium pH.
