# Supplementary material for: A membrane protein of the rice pathogen Burkholderia glumae required for oxalic acid secretion and quorum sensing
Source: Mol Plant Pathol. 2023 Jul 10;24(11):1400–13. doi: 10.1111/mpp.13376 (PMC10576180; doi:10.1111/mpp.13376)
Supplement: Supplementary file 3 — Figure S3. Virulence of Burkholderia glumae 336gr‐1 and ΔobcAB as determined using onion slices. (a) The area of maceration is a measure of the virulence for each strain (Iqbal et al., 2021). Onion slices were infected with 5 × 10^9^ cells of B. glumae 336gr‐1 or ΔobcAB in 10 μL. As a control, 10 μL of sterile MH2 medium was used. The infected onion slices were incubated at 30°C for 4 days in a humid chamber. (b) Area of maceration (cm2) of onion slices produced by B. glumae strains. The bar graph shows the results for six replicates. ***p < 0.001. [file MPP-24-1400-s005.docx]

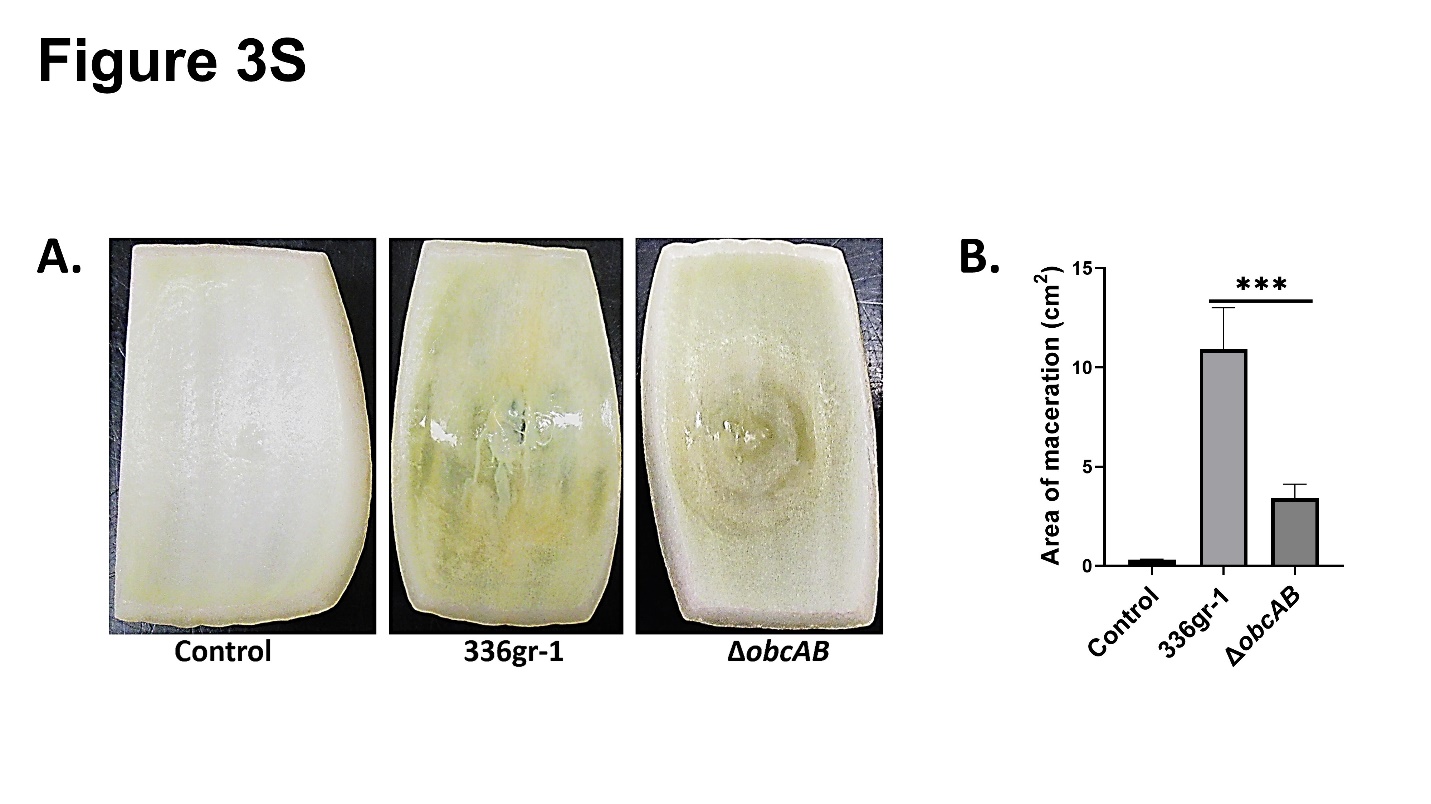

**Figure S3.** Virulence of *B. glumae* 336gr-1 and Δ*obcAB* using onion slices. (A) The area of maceration is a measure of the virulence for each strain (Iqbal *et al.*, 2021). Onion slices were infected with 10 μl of 5 x 10^9^ cells of *B. glumae* 336gr-1 and Δ*obcAB*. For control, sterile 10 μl MH2 media was added. The infected onion slices were incubated at 30 °C for 4 days in a humid chamber. (B) Area of maceration (cm^2^) produced by *B. glumae* strains. The bar graph shows the results for 6 replicates of onion slices. ***, p<0.001

**Reference**

Iqbal, A., Panta, P. R., Ontoy, J., Bruno, J., Ham, J. H. and Doerrler, W. T. (2021) Chemical or Genetic Alteration of Proton Motive Force Results in Loss of Virulence of Burkholderia glumae, the Cause of Rice Bacterial Panicle Blight. *Appl Environ Microbiol,* **87,** e0091521.
