# Supplementary material for: A membrane protein of the rice pathogen Burkholderia glumae required for oxalic acid secretion and quorum sensing
Source: Mol Plant Pathol. 2023 Jul 10;24(11):1400–13. doi: 10.1111/mpp.13376 (PMC10576180; doi:10.1111/mpp.13376)
Supplement: Supplementary file 4 — Figure S4. Restoration of culture media acidification by Burkholderia glumae ΔobcAB through expression of obcAB. (a, b) Culture medium pH and growth of B. glumae strains. B. glumae obcAB was PCR‐amplified from B. glumae genomic DNA and the PCR product was ligated into NdeI and HindIII restriction sites of expression vector pSCrhaB2, resulting in pSC700 (Table 1). B. glumae 336gr‐1 transformed with control vector pSCrhaB2 (vec) and B. glumae ΔobcAB transformed with control vector pSCrhaB2 (vec) and pSC700 (obcAB) were grown on LB agar containing 50 μg/mL trimethoprim and 0.0005% rhamnose. Equal numbers of cells (5 × 10^7^) were inoculated into 250‐mL culture flasks containing 40 mL of LB broth buffered to 7.0 with 70 mM Tris, 40 μg/mL trimethoprim, and 0.0005% rhamnose and grown at 37°C with shaking for 24 h. After 24 h, 5 mL of bacterial culture was collected to measure pH and cell number. **p < 0.01, ***p < 0.001. [file MPP-24-1400-s002.docx]

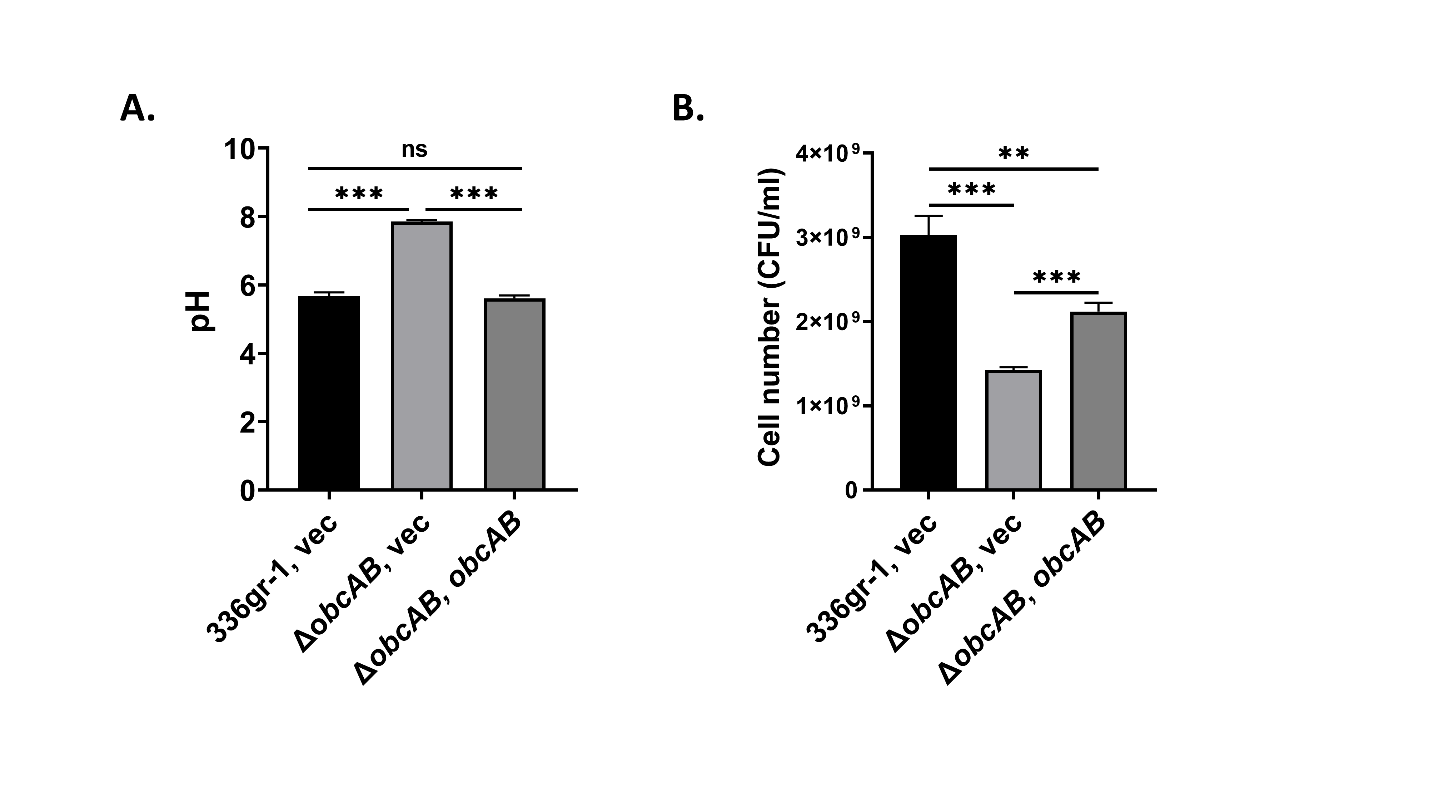


**Figure S4**. **Complementation of culture media acidification in *B. glumae* Δ*obcAB* by expression of *obcAB***. (A and B) Culture medium pH and growth of *B. glumae* strains. *B. glumae obcAB* was PCR amplified from *B. glumae* genomic DNA and the PCR product was ligated into *NdeI* and *HindIII* restriction sites of expression vector pSCrhaB2 resulting in pSC700 (Table 1). *B. glumae* 336gr-1 transformed with control vector pSCrhaB2 (vec) and *B. glumae* Δ*obcAB* transformed with control vector pSCrhaB2 (vec) and pSC700 (*obcAB*) were grown LB agar media containing 50 μg/ml trimethoprim and 0.0005% rhamnose. Equal numbers of cells (5 x 10^7^) were inoculated into a 250 ml culture flask containing 40 ml of LB broth buffered to 7.0 with 70 mM Tris, 40 μg/ml trimethoprim and 0.0005% rhamnose and grown at 37°C with shaking for 24 hours. After 24 hours, 5 ml of bacterial culture were collected to measure pH and cell number. **, p<0.01; ***, p<0.001**.**
