# Supplementary material for: A membrane protein of the rice pathogen Burkholderia glumae required for oxalic acid secretion and quorum sensing
Source: Mol Plant Pathol. 2023 Jul 10;24(11):1400–13. doi: 10.1111/mpp.13376 (PMC10576180; doi:10.1111/mpp.13376)
Supplement: Supplementary file 5 — Figure S5. Oxalic acid levels and acyl‐homoserine lactone (AHL) accumulation during growth of Burkholderia glumae wild type, ΔdbcA, and ΔobcAB in unbuffered LB broth. (a) Oxalic acid production. The inset bar graph shows oxalic acid levels at 6 h. Equal numbers of cells (5 × 10^7^) were inoculated into 250‐mL culture flasks containing 40 mL of either unbuffered or buffered LB broth and grown at 37°C with shaking. Culture supernatants of B. glumae strains were collected by centrifugation at the indicated time points and the oxalic acid level was measured. (b) Acyl‐homoserine (AHL) quantification from culture supernatant of indicated strains. Representative individual wells are shown on the right. N‐octanoyl homoserine (C8‐HSL (10 μM) was added to the positive control, while no C8‐HSL was added to the negative control. Asterisks indicate a statistically significant difference between B. glumae 336gr‐1 and ΔdbcA. *p < 0.05, **p < 0.01, ***p < 0.001. [file MPP-24-1400-s003.docx]

**
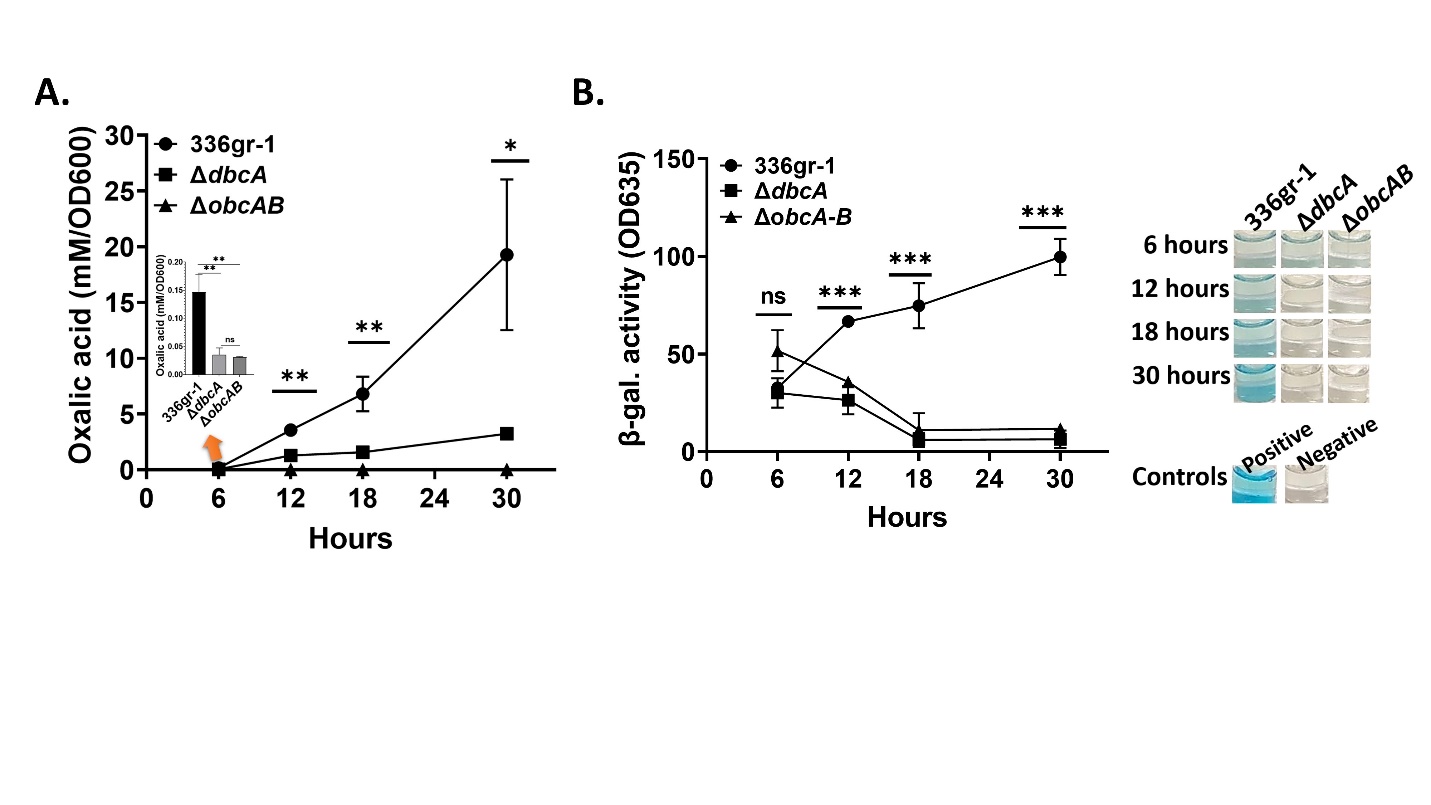
**

**Figure S5. Oxalic acid levels and AHL accumulation during growth of *B. glumae* wild type, Δ*dbcA*, and Δ*obcAB* in unbuffered LB broth*.*** (A) Oxalic acid production. Inset bar graph shows oxalic acid measurement at 6 hours. Equal numbers of cells (5 x 10^7^) were inoculated into a 250 ml culture flask containing 40 ml of either unbuffered or buffered LB broth and grown at 37°C with shaking. Culture supernatants of *B. glumae* strains were collected with centrifugation at the indicated times and oxalic acid was measured. (B) AHLs quantification from culture supernatant of indicated strains. Representative individual wells are shown on the right. C8-HSL (10 µM) was added to the positive control, while no C8-HSL was added to negative control. Asterisks indicate statistical significance between *B. glumae* 336gr-1 and Δ*dbcA.* *, *p*<0.05; **, *p*<0.01; ***, *p*<0.001.
