# Supplementary material for: A membrane protein of the rice pathogen Burkholderia glumae required for oxalic acid secretion and quorum sensing
Source: Mol Plant Pathol. 2023 Jul 10;24(11):1400–13. doi: 10.1111/mpp.13376 (PMC10576180; doi:10.1111/mpp.13376)
Supplement: Supplementary file 6 — Figure S6. Deletion of the Burkholderia glumae 336gr‐1 obcAB operon (bglu_2g18780 and bglu_2g18790). (a) The position of the obcAB operon in the B. glumae 336gr‐1 genome and deletion of the obcAB operon from the genome. The obcAB operon is located between bglu_2g18770 (LysR family transcriptional regulator) and bglu_2g18800 (NADH‐flavin oxidoreductase). The annealing sites for primers A1F, A1R, A2F, A2R, CompFP, and CompRP are shown. Genes are not drawn to scale. Primers A1F, A1R, A2F, and A2R represent Oxalate‐upNEW2FP, Oxalate‐upNEW2RP, Oxalate‐DWN2FP, and Oxalate‐DWN2RP, respectively. (b) 1% agarose gel stained with ethidium bromide showing the confirmation of deletion of the obcAB operon from the B. glumae genome. The PCR amplification using CompFP and CompRP primers produced 2325‐ and 254‐bp DNA fragments for parental strain B. glumae 336gr‐1 and mutant strain B. glumae ΔobcAB, respectively. The 1 kb Plus ladder (Life Technologies) was used to determine the sizes of PCR‐amplified DNA fragments. [file MPP-24-1400-s001.docx]

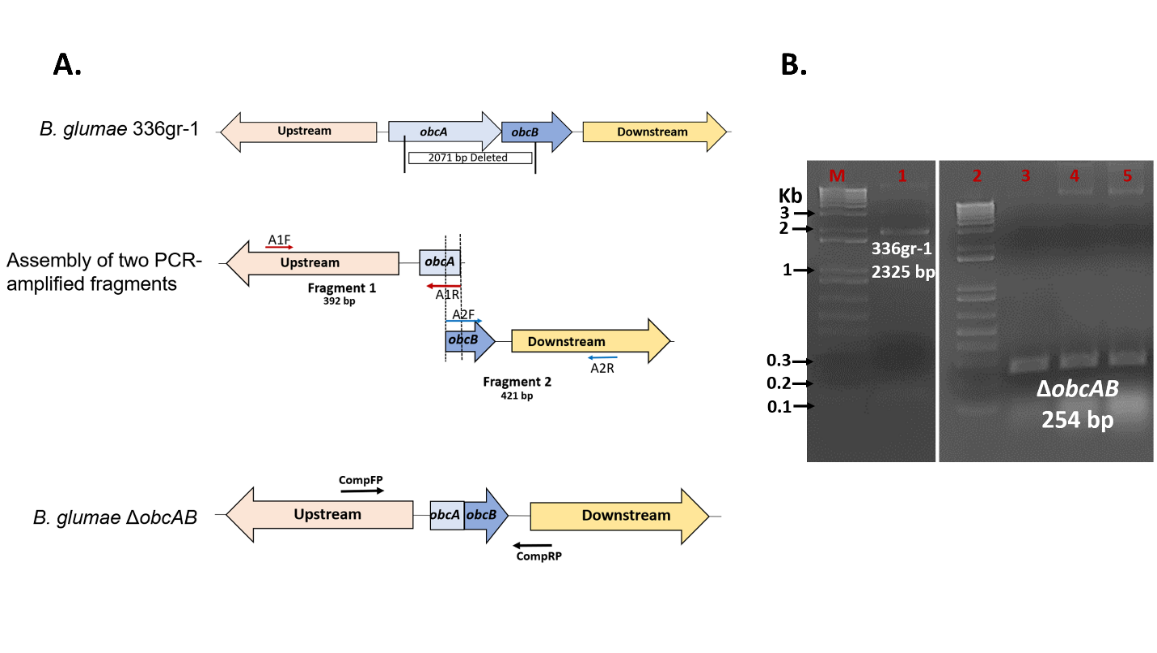


**Figure S6. Deletion of *B. glumae* 336gr-1 *obcAB* operon (*bglu_2g18780*  and *bglu_2g18790*).** (A) The illustration shows the position of *obcAB* operon in the *B. glumae* 336gr-1 genome and deletion of *obcAB* operon from the genome. The *obcAB* operon is located between *bglu_2g18770* (LysR family transcriptional regulator) and *bglu_2g18800* (NADH-flavin oxidoreductase). The annealing sites for primers A1F, A1R, A2F, A2R, CompFP, and CompRP are shown. Genes are not drawn to scale. Primers A1F, A1R, A2F, and A2R represent Oxalate-upNEW2FP, Oxalate-upNEW2RP, Oxalate-DWN2FP, and Oxalate-DWN2RP, respectively (B) 1% agarose gel stained with ethidium bromide shows the confirmation of deletion of the *obcAB* operon from the *B. glumae* genome. The PCR amplification using CompFP and CompRP primers produced 2325 and 254 bp DNA fragments for parental strain *B. glumae* 336gr-1 and mutant strain *B. glumae* Δ*obcAB*, respectively. The 1 kb plus ladder (Life Technologies) was used to determine the sizes of PCR-amplified DNA fragments.
