# Supplementary material for: A membrane protein of the rice pathogen Burkholderia glumae required for oxalic acid secretion and quorum sensing
Source: Mol Plant Pathol. 2023 Jul 10;24(11):1400–13. doi: 10.1111/mpp.13376 (PMC10576180; doi:10.1111/mpp.13376)
Supplement: Supplementary file 7 — Figure S7. Representative standard curve for oxalic acid determination. The absorbance values of 0, 2, 4, 6, 8, and 10 nmol oxalic acid were imported into Microsoft Excel and a standard curve was generated. [file MPP-24-1400-s006.docx]

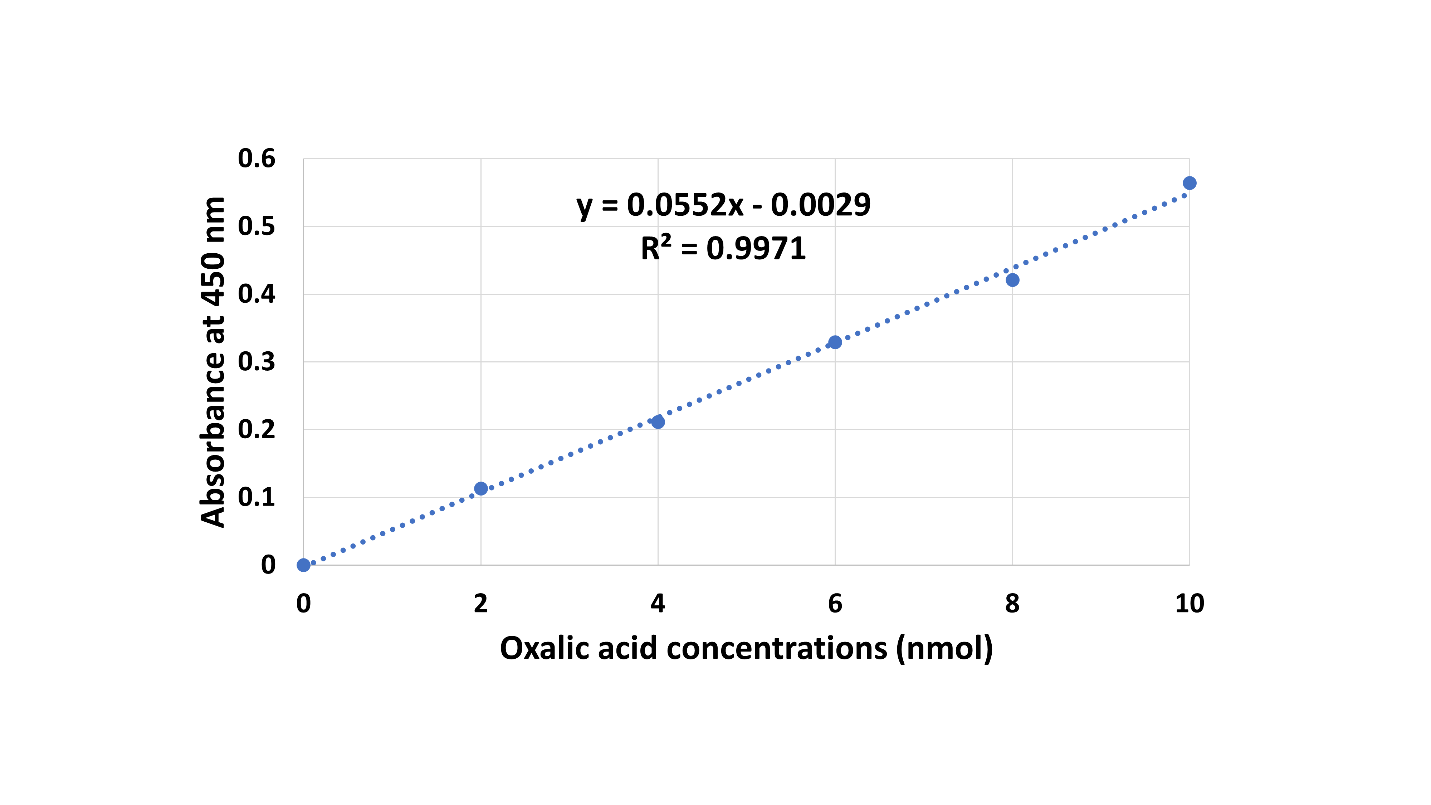


**Figure S7. Representative standard curve for oxalic acid determination.** The absorbance values of 0, 2, 4, 6, 8, and 10 nmol oxalic acid were imported into Microsoft Excel and a standard curve was generated.
