# Supplementary material for: A membrane protein of the rice pathogen Burkholderia glumae required for oxalic acid secretion and quorum sensing
Source: Mol Plant Pathol. 2023 Jul 10;24(11):1400–13. doi: 10.1111/mpp.13376 (PMC10576180; doi:10.1111/mpp.13376)
Supplement: Supplementary file 8 — Table S1. Oligonucleotide primers used in this study. [file MPP-24-1400-s004.docx]

**Table S1**. Oligonucleotide primers used in this study.

| Primer Names | Sequences (5́'→3́)*^a^* |
| --- | --- |
| Oxalate-upNEW2FP | GGATCCGTTTGCATGGGAATGT |
| Oxalate-upNEW2RP | AGAACTCGATTGCCGTGATGTATAGCGATG |
| Oxalate-DWN2FP | CATCACGGCAATCGAGTTCTATGCCGACCA |
| Oxalate-DWN2RP | GGATCCGCTACGAGCTGCATAC |
| Oxalate CompFP | CTGGTCGGCATAGAACTCG |
| Oxalate CompRP | CGAAACATTCGGCGACTTAT |
| obc_Fwr_NdeI | ATAT***CATATG***ATGACATCGCTATACATCACGGC |
| obc_Rv_HindIII | ATAT***AAGCTT***TCACCGCGTCACGCGTACCAGCT |
| **RT-qPCR primers** |  |
| TofI_Fwr_q1 | AACTTTCGTTCACGAGGCA |
| TofI_Rev_q2 | GGGAACACTTCCTGCAACA |
| TofR_Fwr_q1 | ATGGTCAACAGTCCGAACAC |
| TofR_Rev_q2 | CGAGTACTGCTGCTATGGAATC |
| QsmR_Fwr_q1 | GAATCGTCCACGGATGAGATTT |
| QsmR_Rev_q2 | AGGAAGCCTTTGCTCAGTTC |
| ToxJ_Fwr-q1 | CTTCCAGTGTCATCTGGTATCG |
| ToxJ_Rev-q2 | AGCTCTTGAGGAACGTCATTT |
| ToxR_Fwr-q1 | GAAGCGGATCGACCTCAAC |
| ToxR_Rev-q2 | ACATCGACACGCGAAACA |
| ToxA_Fwr-q1 | GTACGACAGCTCGATACGATTC |
| ToxA_Rev-q2 | GCCAGCTCGATCATCTTCTC |
| ToxH_Fwr-q3 | ATATGGCGGTGGATGTTCTC |
| ToxH_Rev-q4 | GTGGTGGTGATCTTCCTGTT |
| Fwr_ObcA-1 | GCCGGCATTATTGAAGAATATGG |
| Rev_ObcA-1 | CGAATGGTTTCCTCGGTGAT |

*^a^* Bold italic text represents restriction enzyme sites.
